# XAPT: Explainable Anomaly-Driven Prediction of Threat Stages in APT Campaigns

**DOI:** 10.1109/access.2025.3636501

**Published:** 2025-11-24

**Authors:** WEI LU, ISSA TRAORÉ, ISAAC WOUNGANG, ERIC BROWN, MARCELO LUIZ BROCARDO, QIAOYAN YU, ORNELLA LUCRESSE SOH

**Affiliations:** 1 Department of Computer Science, Keene State College, Keene, NH 03431, USA; 2 Department of Electrical and Computer Engineering, University of Victoria, Victoria, BC V8P 5C2, Canada; 3 Department of Computer Science, Toronto Metropolitan University, Toronto, ON M5B 2K3, Canada; 4 Department of Computer Science, Dartmouth College, Hanover, NH 03755, USA; 5 Santa Catarina State University (UDESC), Florianópolis, Santa Catarina 88035-901, Brazil; 6 Department of Electrical and Computer Engineering, University of New Hampshire, Durham, NH 03824, USA

**Keywords:** Advanced persistent threats, anomaly detection, score calibration, Bayesian networks, explainable AI

## Abstract

Advanced Persistent Threats (APTs) are long-lived, targeted cyberattacks that progress through multiple stages, characterized by strong stealth and intent. To achieve accurate and interpretable stage-level prediction, we propose XAPT, an eXplainable, anomaly-driven framework for APT campaign analysis. XAPT is centered on three key innovations. First, we derive PCA-based reconstruction errors and transform them into calibrated probabilistic anomaly scores, enabling principled quantification of the event abnormality. Second, these calibrated scores are incorporated into a Bayesian Network-based multiclass classifier for cyber-kill-chain stage inference, capturing uncertainty and inter-feature dependencies. Third, SHAP-based feature attribution reveals how anomaly scores and other features contribute to classification outcomes, offering transparent and analytically friendly explanations. Evaluation of two public datasets shows that XAPT achieves high stage-level detection accuracy while producing actionable feature-level interpretations that support operational analysis. By unifying calibrated anomaly scoring, Bayesian inference, and SHAP explanation, XAPT offers a comprehensive and interpretable solution for advanced threat detection.

## I. INTRODUCTION

Advanced Persistent Threats (APTs) are highly targeted long-term cyberattacks orchestrated by well-funded adversaries with strategic objectives such as sabotage or data exfiltration [1], [2], [3]. Their persistence, stealth, and adaptability allow them to unfold as a series of seemingly benign events that, when considered in isolation, appear harmless but collectively form a damaging campaign. Traditional Intrusion Detection Systems (IDS) and Intrusion Response Systems (IRS), optimized for short-term and opportunistic attacks, often fail against APTs because they rely on homogeneous data sources and cannot capture distributed or time-delayed stages. Consequently, accurate attribution of these stages becomes essential for a timely response, containment, and forensic investigation. However, the long-term, low-and-slow traffic patterns and polymorphic toolchains used by APT adversaries make stage-level prediction extremely challenging.

Recent studies have explored multiple dimensions of APT detection, including kill-chain based rule systems [7], [8], [15], [16], classical machine learning models [17], [18], [19], deep learning architectures [20], [21], [22], provenance-driven correlation frameworks [24], [25], [26], [27], and DNS-based analysis [28], [29], [30]. While these approaches have advanced automated threat defense, they continue to exhibit three limitations (detailed in Section II-B):

- **Weak and indirect use of anomaly information:** Many existing methods rely on static rules [7], [8], [15], [16] or conventional supervised learning approaches [17], [18], [19], where anomaly indicators, such as abnormal flow statistics or reconstruction-error signals, are used only for thresholding or heuristic scoring. These signals are rarely incorporated as first-class probabilistic evidence to support the inference of the progression of the kill chain, limiting their ability to reason over uncertainty.
- **Lack of stage-aware interpretability:** Deep learning-based detectors [20], [21], [22] and provenance-driven models [24], [25], [26], [27] can achieve strong predictive performance, but often function as opaque models. Even when feature-level explanations are attempted, they are rarely aligned with APT stage semantics, diminishing analysts’ ability to justify predictions. Recent surveys [51], [55] highlight that explainability remains an open challenge for prediction at the stage-level of APT.
- **Limited generalization across heterogeneous data sources:** Existing work commonly focuses on single data modalities, such as raw traffic [17], [18], [19], [20], [21], [22], host events [24], [25], [26], [27], or DNS activity [28], [29], [30]. Few systems demonstrate consistent performance when transitioning from network flow logs to SIEM (Security Information and Event Management)-level meta-alerts, and even fewer maintain transparency during this process. Surveys on AI-driven APT detection [51], [52], [53], [54] also suggest that multi-dataset adaptability is rarely evaluated in stage-aware models.

**FIGURE 1.**
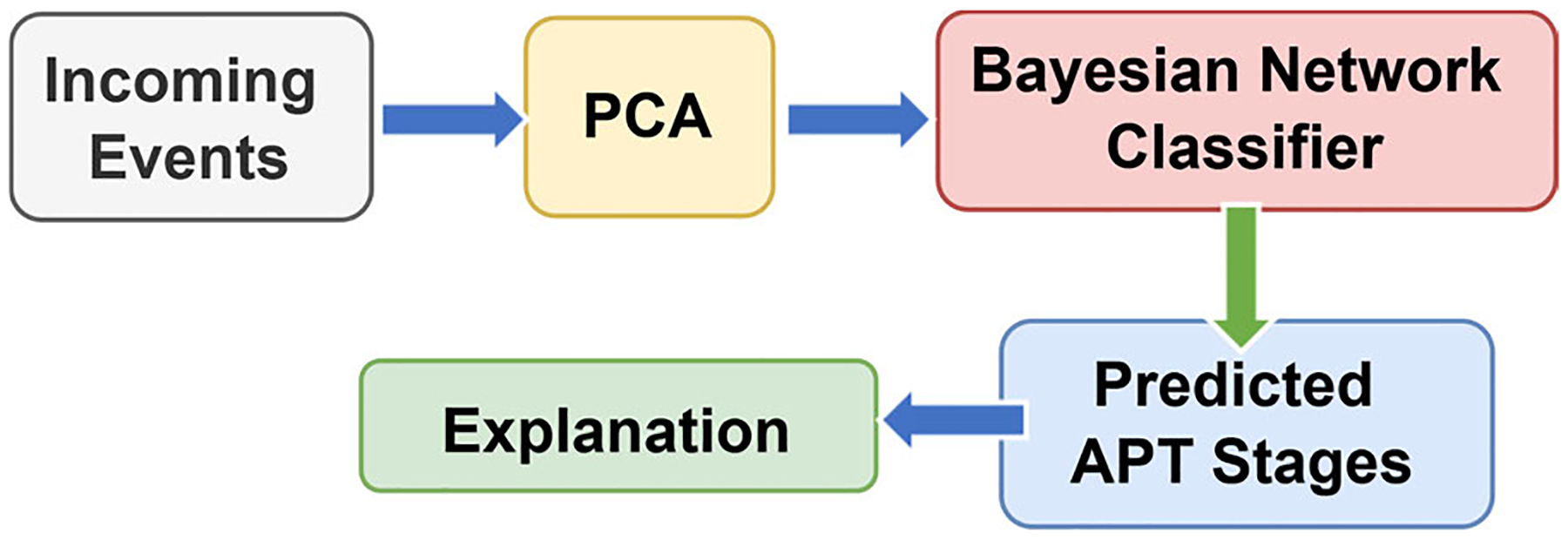
XAPT framework.

These limitations motivate the need for a unified framework that: (1) elevates anomaly evidence into structured probabilistic input; (2) enhances stage-level interpretability; and (3) supports heterogeneous data sources. Addressing these gaps is essential for designing practical and operationally meaningful APT stage prediction systems. As a result, in this paper, we present XAPT, an eXplainable, anomaly-driven framework for predicting APT stages. XAPT integrates three key elements: (1) calibrated anomaly scores derived from PCA reconstruction errors used as central features; (2) Bayesian Network classification, which captures uncertainty and inter-feature dependencies; and (3) SHapley Additive exPlanations (SHAP), which quantify feature contributions and provide transparent, auditable justifications. Figure 1 illustrates the XAPT framework. The pipeline consists of four steps: (1) PCA computes reconstruction errors for each event, which are calibrated into probabilistic anomaly scores; (2) the Bayesian network maps these scores to cyber-kill chain stages; (3) SHAP attributes anomaly scores back to input features, explaining their role in predictions; and (4) the system outputs both the predicted stage and a feature-level explanation.

Compared to existing APT detection solutions, the superiority of the proposed XAPT system includes the following:

- **Anomaly-informed probabilistic inference:** Rather than using reconstruction error from PCA merely as a heuristic, XAPT calibrates anomaly scores into posterior-like probabilities and incorporates them as first-class input to a Bayesian Network. This enables principled stage attribution under uncertainty, outperforming rule-based [7], [8], [15], [16] and conventional ML pipelines [17], [18], [19] that do not leverage probabilistic reasoning.
- **Stage-level explainability:** Unlike deep architectures [20], [21], [22] and graph-based models [24], [25], [26], [27] that produce opaque predictions, XAPT integrates SHAP to quantify feature contributions for every inference, including verifying that calibrated anomaly signals dominate stage classification. This allows analysts to validate why each event is mapped to a specific kill-chain phase.
- **Cross-source adaptability:** While most previous work evaluated only raw traffic [17], [18], [19], [20], [21], [22] or alert-level data [24], [25], [26], [27], XAPT demonstrates consistent stage-mapping performance in both network flow and SIEM meta-alert datasets. This highlights its flexibility in varying data granularities, a capability rarely validated in existing stage-aware APT frameworks [51], [52], [53], [54].

Together, these properties enable XAPT to bridge the gap between stage-level detection accuracy and operational interpretability, key requirements for practical APT defense. Moreover, the experimental validation of XAPT on two public APT datasets demonstrates that it captures attack behaviors at different levels of abstraction, including raw network traffic and SIEM-generated meta-alerts. The experimental results show that XAPT achieves high accuracy in both stage prediction and attack reconstruction while providing transparent and reliable explanations.

In summary, this work makes the following three key contributions:

- **Anomaly-informed Bayesian stage prediction:** We transform PCA-based reconstruction errors into calibrated probabilistic anomaly scores and integrate them into a Bayesian Network, enabling multi-class kill-chain stage attribution with uncertainty modeling.
- **Unified and interpretable detection pipeline:** We incorporate SHAP to quantify feature-level contributions, revealing the dominant role of calibrated anomaly scores and producing transparent, analyst-friendly explanations that strengthen operational decision-making.
- **Cross-dataset effectiveness:** We validate XAPT on both raw network traffic and SIEM meta-alerts, demonstrating consistent stage-level detection performance across heterogeneous data representations.

The remainder of this paper is organized as follows. Section II reviews related work, Section III details the XAPT framework, Section IV presents experimental evaluation, and Section V concludes the paper with directions for future research.

## II. BACKGROUND AND RELATED WORK

### A. BACKGROUND ON APTS

The term *Advanced Persistent Threat* (APT) was first introduced by the US Air Force around 2006 as a general unclassified term used to describe state-sponsored groups capable of launching attacks on military, governmental, and other strategically sensitive assets [1]. Bejtlich provided a detailed breakdown of the components of the term APT:

- **Advanced** refers to adversaries possessing high-level technical skills, including the ability to bypass security defenses by creating custom exploits and, when necessary, leveraging zero-day vulnerabilities.
- **Persistent** indicates that the attacker operates with a specific objective in mind and is willing to sustain a long-term campaign, maintaining access and evading detection as long as necessary to accomplish their mission.
- **Threat** emphasizes that the actor is organized, well funded, and highly motivated, representing a serious and credible danger even to well-defended systems.

Initially, the term APT referred to specific, identifiable threat actors. However, over time, it has evolved into a broader classification used to describe a category of attacks that exhibit the same defining characteristics, namely sophistication, persistence, and strategic intent. The application of the term has also expanded beyond its initial military and governmental context to encompass the private sector, where APTs are frequently associated with the theft of intellectual property and trade secrets. Although some researchers distinguish between general ‘targeted attacks’ and nation-state-sponsored APTs, others use terms such as Advanced Persistent Attack (APA) interchangeably with APT.

**FIGURE 2.**
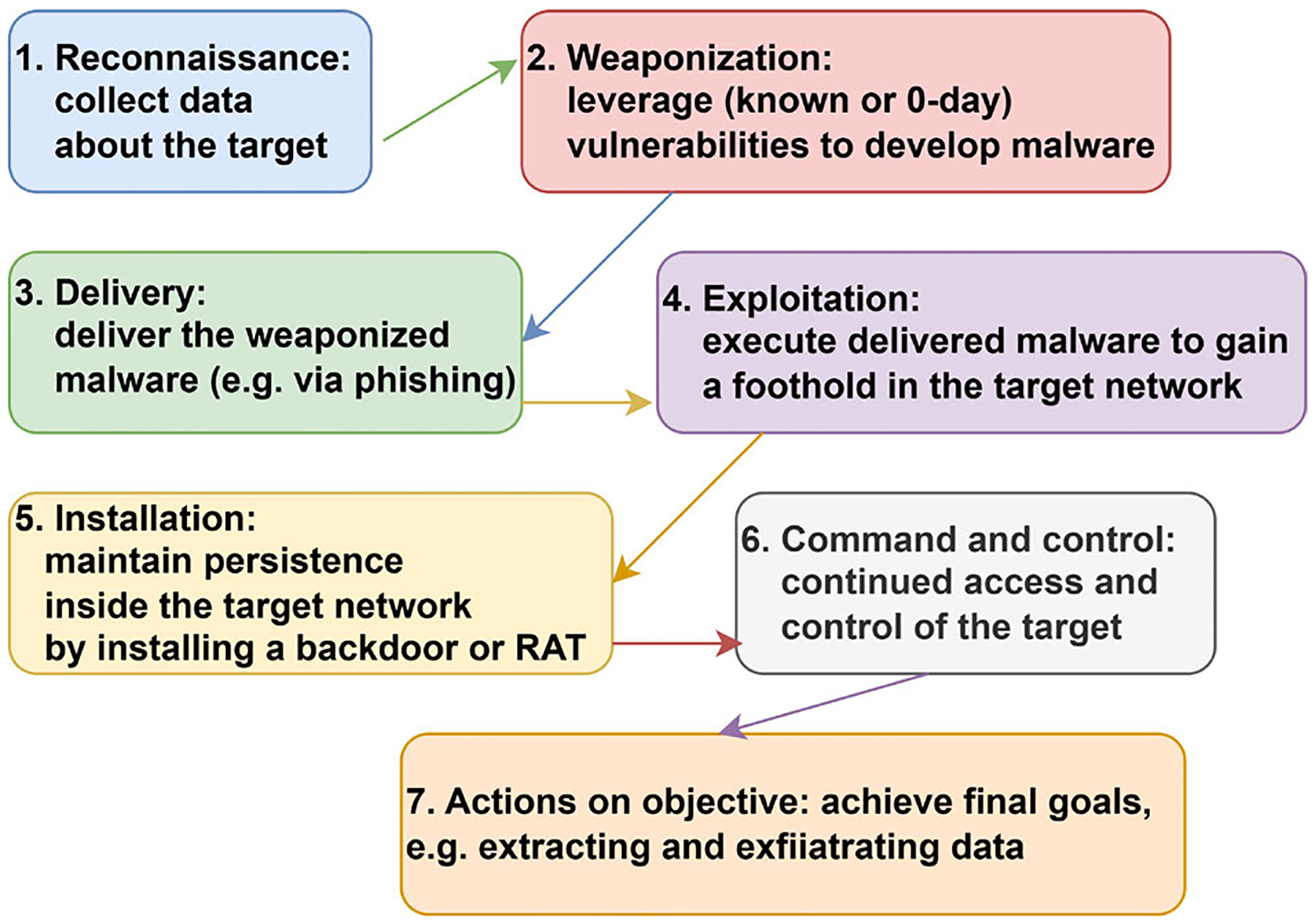
APT cyber-kill chain stages.

Despite their variability and adaptability, APTs tend to follow a broadly consistent life cycle [7], [8], [9], [10], [11], [12]. Although different researchers have proposed slightly varying numbers of stages, their models generally converge on a common structure, as illustrated in Fig. 2. This typical APT life cycle consists of the following phases:

- **Reconnaissance:** This is the initial phase of an APT campaign, in which attackers gather as much intelligence as possible about the target organization, such as its infrastructure, personnel, and relationships, to identify potential vulnerabilities that can be exploited later.
- **Weaponization:** Once viable attack vectors are identified, attackers prepare and test the tools required for exploitation. These tools may include publicly available malware, zero-day exploits purchased from underground markets, or, in more sophisticated campaigns, custom-developed attack tools. The attackers often test these tools in replica environments that mimic the target system, assessing their effectiveness and likelihood of detection or retaliation.
- **Delivery:** In this phase, the attackers transmit the malicious payload to the target. The two most common methods are spear-phishing emails and watering-hole attacks [13]. Web-based attacks often exploit application vulnerabilities, which are commonly found in PDF readers, web browsers, or Microsoft Office software. In cases involving air-gapped networks, physical media such as USB drives may be used as the delivery mechanism. When feasible, attackers can also leverage insider threats, such as disgruntled employees, bribed staff, or planted agents.
- **Exploitation / Foothold Establishment:** After the malicious payload has been delivered and executed successfully, malware attempts to exploit system vulnerabilities to gain control over the compromised machine. This foothold enables the initiation of command-and-control (C&C) communications, often through Remote Access Trojans (RATs), rootkits, or other backdoor mechanisms. Hutchins et al. [7] divide this step into two distinct phases: exploitation and installation.
- **Progression / Command and Control:** Once access is established, attackers begin to expand their presence within the target network. Controlling a single machine is inherently unstable (it can be shut down, wiped, or otherwise become inaccessible) and is often not the ultimate target. As such, adversaries engage in lateral movement to compromise additional hosts and gain access to higher-value systems. This phase typically involves a cyclical process of internal reconnaissance, privilege escalation, and lateral movement. Some researchers (e.g., [9], [11]) further divide this stage into discrete sub-phases to reflect its complexity.
- **Actions on Objectives / Mission Realization:** Once the attackers have reached their intended targets, they proceed to execute their primary mission. This typically involves exfiltrating sensitive data through established command-and-control channels. However, in some cases, such as the Stuxnet attack, the objective may be to sabotage or disrupt critical infrastructure.
- **Covering Tracks:** To avoid detection and attribution, attackers take deliberate steps to erase evidence of their presence. This includes deleting system logs, removing temporary files, and uninstalling malware components that are no longer needed. In practice, these efforts to conceal activity are not limited to the final phase but are carried out continuously throughout the campaign to maintain stealth and prolong access.

Although the APT life cycle provides a useful conceptual framework, effectively detecting and attributing attack stages in practice remains a highly challenging task. Traditional intrusion detection systems often fail to capture the subtle long-term dependencies across stages, motivating a growing body of research into specialized APT detection frameworks.

### B. RELATED WORK ON APT DETECTION

Based on the APT life cycle, prior research has explored various approaches to detect, correlate, and predict advanced persistent threats. These efforts encompass a range of methods, including rule-based approaches, anomaly detection, machine learning, and deep learning models, each with distinct trade-offs in terms of accuracy, scalability, and interpretability.

#### 1) KILL CHAIN-BASED DETECTION

Hutchins et al. [7] introduced the Intrusion Kill Chain framework, which has inspired methods such as those by Giura and Wang [8], Ioannou et al. [15], and Bhatt et al. [16]. These approaches map intrusion events to lifecycle stages and correlate alerts across phases. Although conceptually powerful, many remain rule-based and offer limited predictive capabilities. *In contrast, XAPT builds on the kill chain model but incorporates calibrated anomaly scores and Bayesian inference, providing probabilistic stage attribution beyond static rule sets*.

#### 2) CLASSICAL ML DETECTION

Several works have applied machine learning to host- and network-level data, including decision tree-based behavioral models [17], NetFlow graph analysis [18], and ontology-driven classification frameworks [19]. These methods improve detection accuracy over purely rule-based systems, but still struggle with scalability and feature-level interpretability. *XAPT addresses these limitations by integrating anomaly scores as structured probabilistic features and applying SHAP to reveal their precise role in predictions*.

#### 3) DEEP LEARNING-BASED DETECTION

Hybrid neural architectures such as CNN-LSTM and CNN-MLP [20], autoencoder-based anomaly detectors [21], and reinforcement learning methods [22] have achieved high detection accuracy. However, these models typically operate as opaque models, making it difficult for analysts to understand or audit predictions. *XAPT closes this gap by explicitly combining strong predictive performance with SHAP-based explanations, ensuring that feature contributions remain transparent*.

#### 4) HYBRID AND PROVENANCE MODELS

Frameworks such as RAPTOR [24] and HOLMES [25] correlate events across multiple hosts using graph-based representations of campaigns. Ghafir et al. [26] and Yussuf et al. [27] combined alert correlation with traditional ML classifiers. Although effective in reconstructing attack scenarios, these systems are complex and lack transparent, feature-level explanations. *Our approach complements such correlation methods by producing interpretable, stage-specific predictions that directly highlight the driving features*.

#### 5) DOMAIN- AND DNS-BASED DETECTION

Other approaches target C&C infrastructure through DNS analysis, including random forest classifiers [28], Global Abnormal Forest models [29], and decision tree-based systems [30]. These achieve high precision within their scope, but do not address stage-level attribution of APT campaigns. *By contrast, XAPT provides end-to-end attribution across kill chain stages, going beyond detection of isolated domains or flows*.

Recent work continues to adopt the kill-chain paradigm for APT detection. Ahmed et al. proposed a kill chain-based detection framework to map APT behaviors through stages [27]. Additionally, several surveys have provided systematic perspectives on APT detection. Mat et al. conducted a comprehensive review of APT behaviors and detection strategies [51], while Mutalib et al. presented a review focused on explainable deep learning approaches for APT detection in cybersecurity [55]. Beyond rule-based and anomaly-based models, researchers are increasingly investigating AI-driven methods. Brandao explored the broader role of artificial intelligence in detecting APTs [52], and Arefin et al. systematically compared machine learning algorithms for APT detection, questioning whether superior accuracy alone is sufficient [53]. Venkatakrishnan further proposed a real-time analytics framework that integrates cyber-threat intelligence with provenance graphs for effective APT hunting [54]. More recently, Bahar et al. introduced CONTINUUM [57], which models the evolution of APT using spatial-temporal graph neural networks. Gaudenzi et al. proposed an uncertainty-aware multistage attack classifier [58], leveraging evidential learning to quantify predictive uncertainty. Shaker et al. integrated SHAP into intrusion-detection workflows to improve interpretability along cyber kill-chain phases [59]. These latest studies reflect growing efforts toward temporal reasoning, uncertainty modeling, and explainability; however, they typically do not incorporate calibrated anomaly signals as core probabilistic evidence, nor do they demonstrate robustness across heterogeneous data modalities.

Overall, existing APT detection approaches either emphasize stage-level attribution but lack feature-level interpretability, or achieve high accuracy through advanced ML at the cost of transparency. Few frameworks integrate anomaly scoring, probabilistic reasoning, and explainable AI into a unified solution. Our proposed XAPT system directly addresses this gap by combining calibrated anomaly scores, Bayesian stage classification, and SHAP-based explanations, thus achieving accurate and interpretable APT stage detection across different data sources.

## III. PROPOSED APPROACH

In this section, we provide an overview of our proposed approach, followed by a thorough examination of each step.

### A. APPROACH OVERVIEW

**FIGURE 3.**
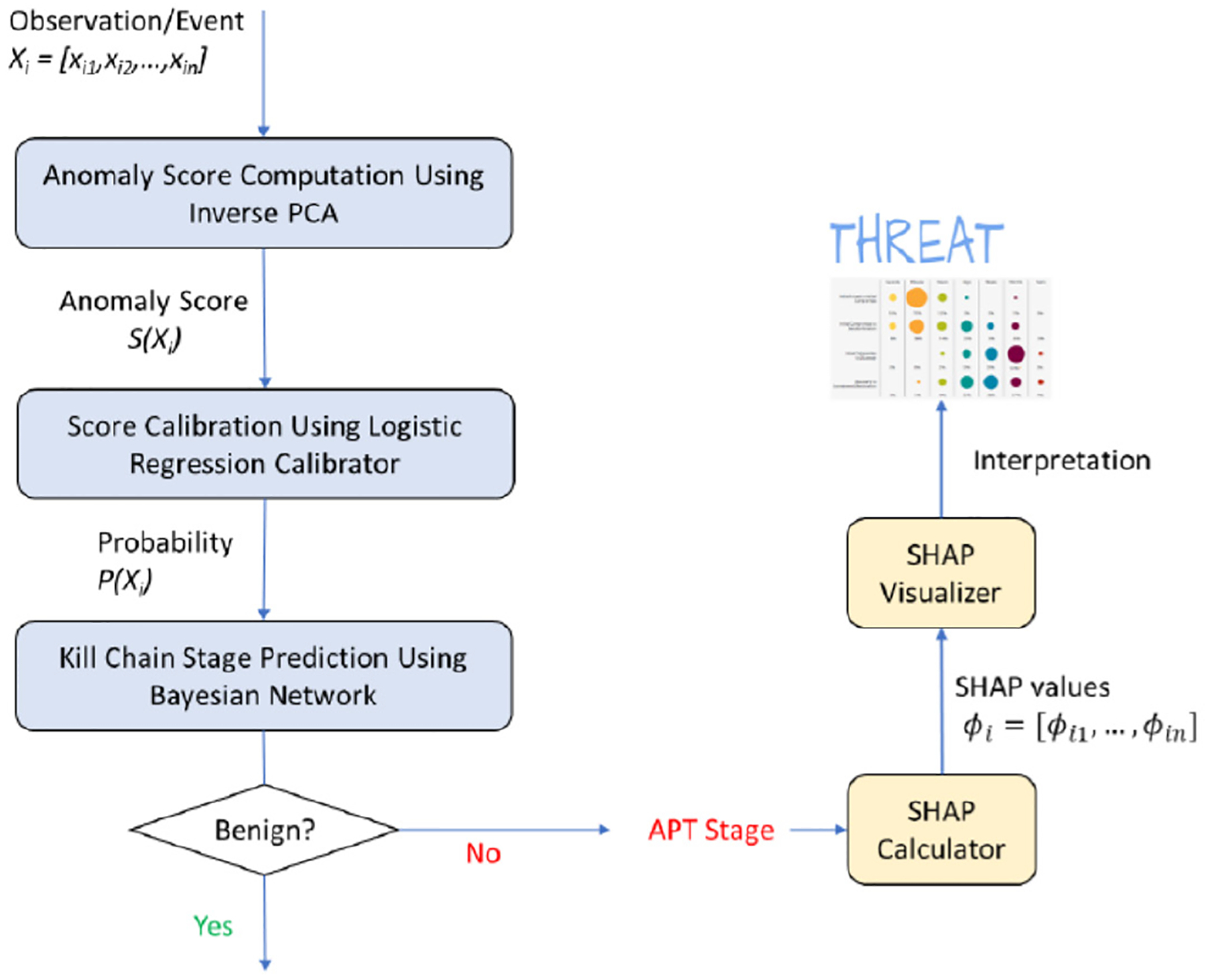
APT detection and stage mapping process in the proposed XAPT framework. The pipeline integrates anomaly scoring via PCA reconstruction, score calibration with logistic regression, Bayesian network classification, and SHAP-based interpretation for feature-level transparency (Adapted from [56]).

Figure 3 illustrates the main steps of our proposed approach. The process begins by computing an anomaly score for each incoming event using inverse Principal Component Analysis (PCA). This score is then transformed into a probability value by using a new score calibration technique introduced in [31]. The calibrated probability is fed into a trained Bayesian Network (BN) classifier to determine whether the event is benign or corresponds to a specific stage in the APT kill chain. Finally, the classifier prediction is passed to an SHAP explainer, which computes SHAP values for input features and presents the results through an interactive visualization component, enhancing interpretability and user insight.

To enhance procedural clarity for reproducibility and implementation, we provide a pseudo-code-style summary of the XAPT framework. The training phase (Algorithm 1) follows a sequence of dimensionality reduction via PCA, anomaly score calibration, optional feature pruning, Bayesian network construction, and SHAP explainer fitting. The runtime inference process (Algorithm 2) applies the trained modules to incoming events in a SIEM and/or SOC setting and, if enabled, generates local explanations for each prediction.

### B. ANOMALY SCORE CALCULATION AND CALIBRATION

Although PCA is traditionally used for dimensionality reduction, in this study, we apply its inverse to compute an anomaly score for each security event based on reconstruction error. The rationale behind using reconstruction error is that a low-dimensional representation captures the essential structure of the data while filtering out noise and irrelevant features. Therefore, the deviation between the original data and its reconstruction reflects potential anomalies, as significant errors indicate behaviors not well represented by the principal components.

**Algorithm 1:** XAPT Training Pipeline

	**Input:** Labeled training dataset $𝒟={\left\{(x_{i},y_{i})\right\}}_{i=1}^{N}$
	**Output:** Trained PCA model, calibration model, Bayesian Network classifier, SHAP explaine
**1**	**Step 1:** Fit PCA to extract latent components from 𝒟
**2**	**foreach** *sample $x_{i}$ in* 𝒟 **do**
**3**	Compute reconstruction error $S(x_{i})$ using inverse PCA
**4**	**Step 2:** Train logistic regression model to calibrate S(⋅) to probability P(⋅)
**5**	**Step 3:** Learn Bayesian Network (BN) classifier using P(⋅) and $y_{i}$ labels
**6**	**Step 4:** Fit SHAP explainer on final model for interpretability
**7**	**return** *Trained PCA, calibrator, BN, SHAP explainer*

A key distinction between our approach and previous PCA-based anomaly detection methods [32] is that we further calibrate the anomaly score as a probabilistic measure. This calibration introduces a more rigorous, objective, and theoretically grounded basis for interpreting anomaly scores, thereby improving decision-making in downstream classification tasks.

To compute the anomaly score, we first apply PCA to the input data and then reconstruct it using the inverse PCA transformation [33]. The score is defined as the deviation between the reconstructed data and the original input, representing the extent of the anomaly.

**Algorithm 2:** XAPT Online Inference for Stage Prediction

	**Input:** Incoming event x; Trained PCA, calibrator, BN, SHAP
	**Output:** Predicted stage label $\hat{y}$; SHAP explanation ϕ
**1**	**Step 1:** Compute anomaly score S(x) using inverse PCA
**2**	**Step 2:** Transform S(x) into calibrated probability P(x) using logistic regression
**3**	**Step 3:** Predict APT stage $\hat{y}$ via Bayesian Network using P(x)
**4**	**Step 4:** Compute SHAP explanation ϕ=SHAP(x)
**5**	**return $\hat{y},\phi$**

Consider an observation or event $x_{i}$ represented in an n-dimensional feature space as $x_{i}={\left[x_{ij}\right]}_{1\leq j\leq n}$, where $x_{ij}$ denotes the $j^{\text{th}}$ feature of $x_{i}$. Given a training set consisting of d observations, let A denote the corresponding data matrix:

$$A={\left[x_{ij}\right]}_{1\leq i\leq d,1\leq j\leq n}\in R^{d\times n}.$$

We apply PCA to the data using the following steps: standardization, computation of the covariance matrix, singular value decomposition (SVD), and projection onto a lower-dimensional subspace.

To begin, the data matrix A is normalized to obtain the standardized data matrix $\tilde{A}$, calculated as follows:

$$\tilde{A}\left[i,j\right]=\frac{A[i,j]-\mu_{i}}{\sigma_{i}},$$

where $\mu_{i}$ and $\sigma_{i}$ denote the mean and standard deviation of the $i^{\text{th}}$ feature across all observations.

Here, 1≤i≤d and 1≤j≤n. The parameter $\mu_{i}$ represents the mean of the $i^{\text{th}}$ row in the data matrix and is calculated as:

$$\mu_{i}=\frac{1}{n}\sum_{1\leq j\leq n}x_{ij},$$

while the standard deviation $\sigma_{i}$ is given by:

$$\sigma_{i}=\frac{1}{n}\sqrt{\sum_{1\leq j\leq n}\left(x_{ij}^{2}-\mu_{i}^{2}\right)}.$$

The key advantage of working with the normalized data matrix $\tilde{A}$ instead of the original matrix A is that normalization helps eliminate bias by ensuring that each entry ${\tilde{x}}_{ij}=\tilde{A}[i,j]$ falls within the range [0, 1]. Once the data are normalized, we compute the covariance matrix $\Sigma \in R^{n\times n}$ as:

(1) $$\Sigma ={\tilde{A}}^{T}\tilde{A}.$$

The covariance matrix Σ is then used to estimate the top p eigenvalues ${\left\{\lambda_{j}\right\}}_{1\leq j\leq p}$ and their associated eigenvectors. Let $V={\left(V_{j}\right)}_{1\leq j\leq p}\in R^{n\times p}$ denote the matrix of eigenvectors and $U={\left(U_{j}\right)}_{1\leq j\leq p}\in R^{n\times p}$ be the projection matrix. The eigendecomposition of Σ is given by:

(2) $$\Sigma =VDU^{T}$$

where $D\in R^{p\times p}$ is a diagonal matrix containing the top p eigenvalues ${\left\{\lambda_{j}\right\}}_{1\leq j\leq p}$.

Given a data vector $x_{i}={\left[x_{ij}\right]}_{1\leq j\leq n}$, PCA transforms it into a reduced-dimensional representation $y_{i}={\left[y_{ij}\right]}_{1\leq j\leq p}$, where p≤n. The projection is computed as:

$$y_{i}=U^{T}x_{i}.$$

To approximate the original high-dimensional measurement, the inverse PCA transformation is applied as:

$$x_{i}^{*}=Uy_{i}.$$

The anomaly scores are computed by summing the squared differences between the original and reconstructed data. A larger discrepancy indicates a higher likelihood that the observation is anomalous. To normalize these scores, a minmax scaling technique is applied, ensuring that the final score falls within the range [0, 1].

The anomaly score for observation $x_{i}$ is defined as follows:

(3) $$\text{score}\left(x_{i}\right)=\sum_{j=1}^{n}{\left(x_{ij}-x_{ij}^{*}\right)}^{2}$$

Equation (4) is used to scale these scores:

(4) $$\text{scõre}\left(x_{i}\right)=\frac{\text{score}\left(x_{i}\right)-{\text{score}}_{\text{min}}}{{\text{score}}_{\text{max}}-{\text{score}}_{\text{min}}}$$

where ${\text{score}}_{\text{max}}$ and ${\text{score}}_{\text{min}}$ are the maximum and minimum values of the scores obtained from the training data.

To enable more rigorous decision-making, we convert the anomaly scores into calibrated probability scores. Calibration refers to the process of transforming the output of a classifier into a posterior probability (PP) estimate [31]. Depending on the application, this can be performed using either single-score calibration or multi-score calibration. The key distinction between the two lies in the number of classifier outputs used: Multi-score calibration aggregates the output of multiple classifiers for a single observation, while single-score calibration relies on the output of a single classifier.

Several calibration techniques are available, including logistic regression, isotonic regression, binning, and the PROPROC method. These techniques vary in their modeling assumptions and underlying mechanisms, such as modeling posterior probability directly, using likelihood functions, or using likelihood ratios. Among them, logistic regression is particularly well-suited for multi-score calibration due to its flexibility in handling feature vectors of arbitrary size.

The posterior probability (PP) for an observation $x_{i}$ can be calculated as follows:

(5) $$P\left(\text{class}|\text{scõre}\left(x_{i}\right)\right)=\text{Likelihood}\left(\text{scõre}\left(x_{i}\right)|\text{class}\right)\times \text{prior}(\text{class})$$

Here, given a particular score, the PP is the conditional probability of the estimated class. The likelihood and prior are defined similarly. The prior distribution captures uncertainty and can be modeled using various distributions, such as normal or Bernoulli distributions. Equation (5) can be solved using a convex optimization problem:

(6) $$\text{clâss}=\text{arg}\;\underset{\text{class}}{\text{max}}\;P\left(\text{class}|\text{scõre}\left(x_{i}\right)\right)$$

(7) $$=\text{arg}\;\underset{\text{class}}{\text{max}}\;\text{log}\;P\left(\text{class}|\text{scõre}\left(x_{i}\right)\right)$$

Given a score, the estimated class clâss is the one with the maximum posterior probability. Because the logarithm is a monotonically increasing function, maximizing the log-probability (Equation 7) is equivalent to maximizing the probability (Equation 6), and helps prevent numerical underflow and improves computational efficiency.

The performance of the calibrator was evaluated using two metrics. The first is the mean squared error (MSE), which measures the squared difference between the true posterior probability and its calibrated estimate. The second is the Brier score, which quantifies the squared difference between the actual class label and the calibrated probability score. Both metrics reflect the accuracy and reliability of the calibration process, with lower values indicating better calibration performance.

### C. ATTACK CLASSIFICATION AND STAGE PREDICTION

Given an observation, we perform classification using a multi-class Bayesian network. A Bayesian network is a probabilistic graphical model in which nodes represent discrete or continuous variables, and edges denote probabilistic dependencies between them. This model is particularly effective for handling inputs characterized by high sensitivity and uncertainty, as it enables the updating of beliefs about unknown parameters based on observed data.

The Bayesian network is structured as a directed acyclic graph (DAG), where each node is associated with a conditional probability distribution given its parent nodes. This framework captures the joint probability distribution on all variables in the graph.

Applying the Bayes rule, we transform the scoring function in Equation 7 into conditional probabilities with interpretable and actionable properties. The conditional probability of a class given an observation is defined as follows:

Using Bayes’ theorem, the conditional probability of a class given a calibrated anomaly score can be expressed as:

(8) $$p\left(\text{class}|\text{scõre}\left(x_{i}\right)\right)=\frac{p\left(\text{scõre}\left(x_{i}\right)|\text{class}\right)\cdot P(\text{class})}{P\left(\text{scõre}\left(x_{i}\right)\right)}$$

Since the denominator $P\left(\text{scõre}\left(x_{i}\right)\right)$ is independent of the class, it can be treated as a constant. Thus, Equation 8 can be rewritten as:

(9) $$P\left(\text{class}|\text{scõre}\left(x_{i}\right)\right)\propto P\left(\text{scõre}\left(x_{i}\right)|\text{class}\right)\cdot P(\text{class})$$

Equation 9 implies that the posterior probability is proportional to the product of the likelihood and the prior. Substituting Equation 9 into the logarithmic transformation optimization objective, we obtain:

(10) $$\text{clâss}=\text{arg}\;\underset{\text{class}}{\text{max}}\;\text{log}\;P\left(\text{class}|\text{scõre}\left(x_{i}\right)\right)$$

(11) $$=\text{arg}\;\underset{\text{class}}{\text{max}}\;\text{log}\;P\left(\text{scõre}\left(x_{i}\right)|\text{class}\right)+\text{log}\;P(\text{class})$$

where P(class) is the prior probability of the class, and $P\left(\text{scõre}\left(x_{i}\right)\right)$ is the marginal probability of the score.

We used the bnlearn library (Bayesian Network Learning) to train our model and make predictions. This library supports both structure learning, which identifies relationships among variables in the data, and parameter learning, which estimates the associated conditional probabilities. Structure learning is performed using DAG (Directed Acyclic Graph) search algorithms such as Chickering and K2, while parameter learning relies on the maximum likelihood estimator. In our work, we applied the Gaussian Naive Bayes classifier, which assumes that the feature likelihoods follow a normal distribution according to the following formula:

(12) $$P\left(\text{class}|\text{sc}\overset{\sim }{o}\text{re}\left(x_{i}\right)\right)=\frac{1}{\sqrt{2\pi \sigma_{\text{sc}\overset{\sim }{o}\text{re}\left(x_{i}\right)}^{2}}}\times \text{exp}\left(-\frac{{\left(\text{class}-\mu_{\text{sc}\overset{\sim }{o}\text{re}\left(x_{i}\right)}\right)}^{2}}{2\sigma_{\text{sc}\overset{\sim }{o}\text{re}\left(x_{i}\right)}^{2}}\right)$$

where the mean $\mu_{\text{sc}\overset{\sim }{o}\text{re}\left(x_{i}\right)}$ and standard deviation $\sigma_{\text{sc}\overset{\sim }{o}\text{re}\left(x_{i}\right)}$ are estimated using the maximum likelihood.

Given an observation, the classifier is employed both for basic attack classification (i.e., distinguishing benign vs. attack samples) and for attack stage prediction.

Let

$$L=\left\{L_{r}|0\leq r\leq m\right\}$$

denote the set of stages, where $L_{0}$ corresponds to benign or legitimate activities, and the remaining stages (1≤r≤m) represent the kill-chain attack stages. Intuitively, the higher the cyber kill-chain stage, the more dangerous the activity, and thus the greater the anomaly score. Based on this intuition, each stage $L_{r}$ can be characterized by a calibrated score interval.

$$\left[p{\left(L_{r}\right)}_{\text{min}},\;p{\left(L_{r}\right)}_{\text{max}}\right].$$

Accordingly, an observation $x_{i}$ is assigned to stage $L_{r}$ if

$$p{\left(L_{r}\right)}_{\text{min}}\leq \text{calibrat}\overset{\sim }{e}\text{d\_score}\left(x_{i}\right)\leq p{\left(L_{r}\right)}_{\text{max}}.$$

By treating each stage as a distinct class, stage prediction for a given observation $x_{i}$ is formulated as a multi-class classification task, where the predicted stage corresponds to the class with the maximum calibrated probability:

(13) cla^ss=fxi=argmaxLr∈LlogPLr∣sco~rexi

where f denotes the Bayesian network prediction model.

### D. SHAP-BASED EXPLAINER

SHAP (SHapley Additive exPlanations) leverages a game-theoretical approach to explain the predictions of machine learning models. By incorporating Shapley values from cooperative game theory, SHAP provides a framework that connects optimal credit distribution with localized interpretations. Shapley values allocate credit or blame among contributing factors by calculating the average contribution of each factor to a prediction. SHAP quantifies this by assigning each feature a SHAP value which reflects the average contribution of that feature to moving the prediction from the mean value to the actual prediction. This enables a clear understanding of which features have a significant influence on a specific prediction, providing valuable insights into the decision-making process of a model.

For our Bayesian network prediction model f (defined in Equation 13), given an observation $x_{i}$ represented in a n-dimensional feature space as $x_{i}={\left[x_{ij}\right]}_{1\leq j\leq n}$, where $x_{ij}$ denotes the $j^{\text{th}}$ feature of $x_{i}$, the SHAP value $\phi_{ij}(f)$ for this feature is calculated as follows:

(14) $$\phi_{ij}=\sum_{S\subseteq N\backslash \left\{x_{ij}\right\}}\frac{|S|!(n-|S|-1)!}{n!}\left[f_{x}\left(S\cup \left\{x_{ij}\right\}\right)-f_{x}(S)\right]$$

where:

- N is the set of features;
- S is a subset of features excluding feature $x_{ij}$;
- |S| denotes the number of features in set S;
- $f_{x}\left(S\cup \left\{x_{ij}\right\}\right)$ is the prediction of the model when the feature $x_{ij}$ is added to the subset S;
- $f_{x}(S)$ is the prediction of the model when using feature subset S.

The equation demonstrates that the difference between the model prediction for a specific instance $x_{i}$ and the average model prediction is fairly distributed among the features, represented by their corresponding SHAP values. Our SHAP-based explainer consists of two main components: a *SHAP calculator*, which computes SHAP values for each feature to quantify their contribution to the prediction, and a *SHAP visualizer*, which provides intuitive visualizations to support interpretation. The visualizer illustrates how each feature affects the output of the model by showing the progression from the base value (that is, the prediction of the model without that feature) to the final predicted result. This visual aid is designed to improve interpretability for security analysts and threat hunters.

## IV. EXPERIMENTAL EVALUATION

To assess the performance of our model, we conducted experiments on two publicly available benchmark datasets for APTs. The first dataset is the DAPT2020 dataset proposed by Myneni et al. [34], and the second is the meta-alert dataset for APT detection released by Tork and Khosravi [35]. The DAPT2020 dataset enables the evaluation of both attack detection and stage prediction, whereas the meta-alert dataset is used exclusively for stage prediction, as it only contains attack data represented in the form of meta-alerts. In the following sections, we present and analyze the evaluation results obtained from these two datasets.

### A. PERFORMANCE METRICS

To evaluate our approach, we generated a confusion matrix for each dataset and used it to compute standard performance metrics, including precision, recall, F1 score, and accuracy.

Minimizing false positives is crucial to prevent alarm fatigue, as excessive false alarms may cause security analysts to disregard genuine alerts. Fewer false positives indicate higher precision. Similarly, high recall is desirable because it reflects the system’s ability to detect more attacks correctly.

Accuracy measures the system’s general capability to correctly classify both benign and malicious observations. It is a reliable performance indicator when false positives and false negatives incur similar costs or when the dataset is balanced. However, in cases where the cost of false positives and false negatives differs significantly, the F1 score is more appropriate. The F1 score balances precision and recall, making it particularly suitable for imbalanced datasets, a common characteristic of intrusion detection benchmarks.

To compute the metrics per class, we used the confusion matrix to determine the number of true positives, false positives, and false negatives. For a given class ${\text{Class}}_{i}$, the number of true positives $TP_{i}$ corresponds to the diagonal entry (i,i). The number of false positives $FP_{i}$ and false negatives $FN_{i}$ correspond to the misclassified samples in the same column and row, respectively. The number of true negatives $TN_{i}$ is obtained by summing all remaining cells of the matrix.

The per-class metrics are computed as follows:

(15) $${\text{Precision}}_{i}=\frac{TP_{i}}{TP_{i}+FP_{i}}$$

(16) $${\text{Recall}}_{i}=\frac{TP_{i}}{TP_{i}+FN_{i}}$$

(17) $$F1_{i}=\frac{2\cdot {\text{Precision}}_{i}\cdot {\text{Recall}}_{i}}{{\text{Precision}}_{i}+{\text{Recall}}_{i}}$$

(18) $${\text{Accuracy}}_{i}=\frac{TP_{i}+TN_{i}}{TP_{i}+TN_{i}+FP_{i}+FN_{i}}$$

The macro-average of each metric is calculated as the arithmetic mean of the per-class values. Macro averages are particularly useful for imbalanced datasets, where all classes are treated with equal importance. To account for the varying contributions of individual classes, weighted averages are also reported by computing the mean of per-class values weighted by the number of actual occurrences of each class in the dataset.

Depending on the size of the data set, different strategies can be applied to split the data. Using too little training data can lead to high variance and unreliable cross-validation results. A common recommendation is to select a split that balances variance and bias. With sufficient data, the differences between splits such as 80:20, 70:30, or 90:10 are minimal. However, 80:20 is often considered a suitable starting point, unless computational constraints suggest otherwise [36].

We experimented with three classifiers trained using different split ratios and evaluated their performance on the validation data. The best performance was obtained with the 80:20 split. Consequently, we report results using the model trained with 80% of the data and tested on the remaining 20%.

### B. EVALUATION USING THE DAPT2020 DATASET

#### 1) DATASET OVERVIEW

The DAPT2020 dataset [34] simulates APT behavior in a cloud environment. It contains public network traffic data collected over five days, where day 1 corresponds to legitimate activities and days 2–5 correspond to various APT attack vectors. These attack vectors include data exfiltration, network scanning, account discovery, brute-force attacks, web vulnerability scanning, SQL injection, CSRF, malware download, backdoor access, and command injection.

Each day covers both private and public network data, stored in 10 CSV and PCAP files. The dataset comprises 85 features, with activity and stage information included for labeling purposes. It contains both benign data and attack data corresponding to four primary stages of APT: reconnaissance, foothold establishment, data exfiltration, and lateral movement. In total, the dataset comprises 17,339 samples, which are unevenly distributed across the classes, resulting in a highly imbalanced dataset.

A subset of the 85 features included in DAPT2020 is illustrated in Table 1.

#### 2) EVALUATION RESULTS

We began by concatenating the ten CSV files to form a single dataset. During preprocessing, we verified that the dataset contained no missing or duplicate values.

The data were then divided into training and testing subsets, with 80% of the data used for training and 20% reserved for testing. A single-score calibration approach was applied. The main advantage of this calibration method is that the original and estimated performance remains consistent after calibration.

We used two types of calibrators: logistic regression with and without Platt’s scaling. As expected, Platt’s method, initially developed for SVM, outperformed standard logistic regression because it reduces bias and provides probability distributions between classes by converting the outputs of a non-probabilistic model. Table 2 illustrates sample observations with scores before and after calibration using Platt’s scaling, while Table 3 shows calibrated scores and their corresponding stages.

As anticipated, the probability values increased progressively with higher attack stages.

For stage prediction, we trained a multi-class classifier using the *Stage* feature as labels. The data set consists of five stages, namely benign, reconnaissance, foothold establishment, lateral movement, and data exfiltration, labeled 0 through 4. By training and testing the classifier, we obtained the confusion matrix presented in Table 4.

Table 5 provides a per-class breakdown of the performance metrics computed from the confusion matrix, while Table 6 summarizes the global results, including both macro and weighted average metrics.

Our approach achieved high accuracy across all stages of the DAPT2020 dataset, ranging from just over 81% for stage 0 (benign) to more than 97% for stage 2. However, recall and F1 scores varied considerably between the different stages. Although stages 0, 1, and 2 achieved high recall and F1 scores, the performance for stages 3 and 4 was noticeably lower. This decline is primarily due to the small number of samples in these classes; in particular, class 4 contains only three samples, which is insufficient for effective training of the classifier. However, as summarized in Table 6, the weighted averages, which account for the class imbalance, produced relatively strong results, with accuracy, precision, recall, and F1 scores of 84.59%, 73.39%, 95.27%, and 81.79%, respectively.

### C. EVALUATION USING THE META-ALERTS DATASET

#### 1) DATASET OVERVIEW

The semi-real meta-alert dataset [35] was created by randomly injecting a set of APT attack meta-alerts into a baseline dataset of meta-alerts that was initially free of APT activity. This baseline dataset was generated over 3 days by a SIEM system deployed in a corporate environment with 250 hosts. The injected APT meta-alerts were derived from traces of real-world APT attack scenarios executed over a 7-day period, covering a diverse range of attack vectors, including reflective loading, in-memory module loading, and web-shell functionality. The simulated APT campaigns involved multiple activities, including drive-by downloads, backdoor deployment, privilege escalation, reconnaissance, data exfiltration, and footprint concealment.

The final dataset contains 118,465 samples distributed almost evenly at the different stages (approximately 25% each). It is organized into 250 text files, each corresponding to a single host in the company network. Every meta-alert record includes seven attributes: alert creation time, alert severity, source address, source port, target address, target port, and the associated intrusion kill chain step. The meta-alerts were grouped into four primary APT kill chain phases: (1) reconnaissance and delivery, (2) exploitation, (3) operation, and (4) data collection with exfiltration.

A detailed list of meta-alert features and their data types is provided in Table 7.

#### 2) EVALUATION RESULTS

In addition to the preprocessing step described for the previous dataset, the meta-alert dataset contained text files with eight features, including an additional column with NaN values. This column was removed, and we applied an 80%–20% train–test split to each file prior to concatenation. Afterward, anomaly scores were computed and calibrated before being passed to the Bayesian model.

Table 8 presents the calibration results for representative samples, comparing anomaly scores before and after the calibration step.

As noted earlier, since this dataset contains only attack data (i.e., alerts), it was used exclusively to evaluate the stage prediction model through multi-class classification. The classifier was trained and tested by treating the intrusion kill chain (IKC) step feature as the class label. Four IKC stages were considered: (1) reconnaissance and delivery, (2)exploitation, (3) operation, and (4) data collection with exfiltration. Because there were no benign data, stage 0 was excluded.

Table 9 presents the confusion matrix, showing that the model misclassified only five samples. Table 10 reports the performance metrics for each class, while Table 11 summarizes the overall performance using macro-averaged results across all classes. Weighted averages are omitted as the class distribution is already balanced. In general, the approach achieved consistently high performance, with all metrics exceeding 99%.

Compared to the *DAPT2020* dataset, our approach achieved significantly better performance on the meta-alerts dataset across all metrics and stages. It achieves high accuracy, recall, precision, and F1 scores for all four APT stages. In contrast to *DAPT2022*, meta-alerts is a balanced dataset, with enough samples in each class to properly train a classifier. This is a possible explanation for the performance difference between the two datasets. Another possible reason is that the two datasets operate at different levels of abstraction: raw data versus alerts. This could be an indication that our approach may be better suited for use as an extension for SIEM or in environments where there is a layer of detection systems that generate and feed alerts to it for further classification.

Table 12 compares the results obtained in our work with the performance of previous works on APT detection. Although our performance with *DAPT2020* is on par with most existing works, our results with the meta-alert dataset outperform them.

To further demonstrate the competitiveness of XAPT, we also benchmark our approach against three recent works from 2025: CONTINUUM [57], an uncertainty-aware attack stage classifier [58], and an explainable IDS tailored to APT kill chains [59]. Table 13 compares these approaches in terms of stage awareness, explainability, data granularity, cross-source evaluation, and probabilistic modeling.

As shown in Table 13, 10APT is the only method among these recent works that simultaneously provides stage-level attribution, feature-level (SHAP) explanations, explicit probabilistic reasoning, and validated cross-source performance (raw flows and SIEM meta-alerts).

### D. MODEL EXPLAINABILITY WITH SHAP

In this section, we apply SHAP-based explainability to understand the model’s behavior. It is important to note that this analysis is conducted only on the DAPT2020 dataset. The reason is twofold: (1) DAPT2020 provides fine-grained flow-level features (e.g., flow duration, packet rates, flag counts), which are continuous and diverse, making them well suited for feature attribution analysis; (2) the meta-alert dataset, as shown in Table 14, contains only a small set of high-level attributes (e.g., source/target addresses, port numbers, and impact severity), most of which are categorical or sparse. Applying SHAP to such limited features would trivially assign importance to one or two categorical fields without yielding substantive interpretability. For this reason, SHAP analysis is restricted to DAPT2020, where feature-level contributions can be meaningfully interpreted.

Before applying explainability techniques, we first addressed data set biases that could confuse interpretation. The timestamp feature exhibited strong temporal bias, since malicious activities appeared only on specific days and at certain hours. Similarly, the distribution of the source port was skewed, i.e., no attacks were observed on reserved ports, while the lateral movement class relied exclusively on ports 55249 and 55250. The distribution of the destination port also showed limited variance, with most records concentrated in a single port. To avoid misleading attributions, we excluded these biased features from the explainer.

Next, we performed feature selection to reduce dimensionality, remove noise, and eliminate redundancy. This ensured that the most informative attributes were retained, thereby allowing a model to operate faster, be less prone to overfitting, and become easier to interpret.

Our methodology is split into two sequential stages. First, feature redundancy is addressed by correlation-based pruning. In this stage, we removed 24 redundant features. Redundant features are identified by computing their pairwise correlation:

(19) $$\rho_{ij}=\frac{\text{cov}\left(X_{i},X_{j}\right)}{\sigma_{X_{i}}\sigma_{X_{j}}},\;\forall i\neq j,$$

where $\rho_{ij}$ is the Pearson correlation between features $X_{i}$ and $X_{j},\text{cov}\left(X_{i},X_{j}\right)$ is the covariance, and $\sigma_{X_{i}},\sigma_{X_{j}}$ are the standard deviations of the features [39].

Any pair satisfying

(20) $$\left|\rho_{ij}\right|\geq 0.8$$

is considered redundant. Next, the feature with the higher mutual information (MI) with the target y is retained. Mutual information is defined as

(21) $$MI\left(X_{j};y\right)=\sum_{x_{j}\in X_{j}}\sum_{y\in Y}p\left(x_{j},y\right)\text{log}\frac{p\left(x_{j},y\right)}{p\left(x_{j}\right)p(y)}.$$

Second, we apply Elastic Net regularization for further refinement on the data. Elastic Net merges the sparsity of the LASSO penalty with the stability of Ridge regression, making it more robust for high-dimensional data. The Elastic Net objective function is given by

(22) $$\overset{ˆ}{\beta }=\underset{\beta }{\text{arg}\;\text{min}}\;\left\{-\ell (\beta ;X,y)+\lambda \left(\alpha {\left\|\beta \right\|}_{1}+\frac{1}{2}(1-\alpha ){\left\|\beta \right\|}_{2}^{2}\right)\right\},$$

where ℓ(β;X,y) denotes the logistic log-likelihood, λ>0 controls the overall regularization strength, and α∈[0,1] balances between the LASSO (α=1) and Ridge (α=0) penalties [40].

In our implementation, we set C=0.8, which applies moderately strong regularization, and α=0.5, giving equal weight to the LASSO and Ridge components. The top twenty features were then chosen for our feature set.

This methodology reduced the number of features to the twenty most influential variables. After re-training our model with the new feature set, we produced an accuracy score of 92.32%

To interpret the predictions of the model, we employ SHapley Additive exPlanations (SHAP). SHAP is a framework based on game theory that assigns an importance value to each feature for a single prediction. It utilizes the concept of Shapley values from game theory, where the contribution of each feature to the overall prediction is fairly distributed.

SHAP can be applied at both the local (per sample) and global (dataset level) levels. This explains how specific features influence a prediction’s direction toward or away from a class, relative to the baseline. The baseline is defined by using a subset of the training data while maintaining the overall structure of the data.

#### 1) GLOBAL EXPLANATIONS

To obtain a global view of the importance of the features, we aggregated the magnitudes of the feature attributions across the dataset as in [5]:

(23) $$I_{j}=E_{x}\left[\left|\phi_{j}(x)\right|\right]=\frac{1}{N}\sum_{i=1}^{N}\left|\phi_{j}\left(x_{i}\right)\right|,$$

where $I_{j}$ denotes the global importance of feature $j,\phi_{j}\left(x_{i}\right)$ is the SHAP value attributed to feature j for instance $x_{i},N$ is the total number of instances in the dataset, and $E_{x}[\cdot ]$ represents the empirical expectation (average) over all instances.

**FIGURE 4.**
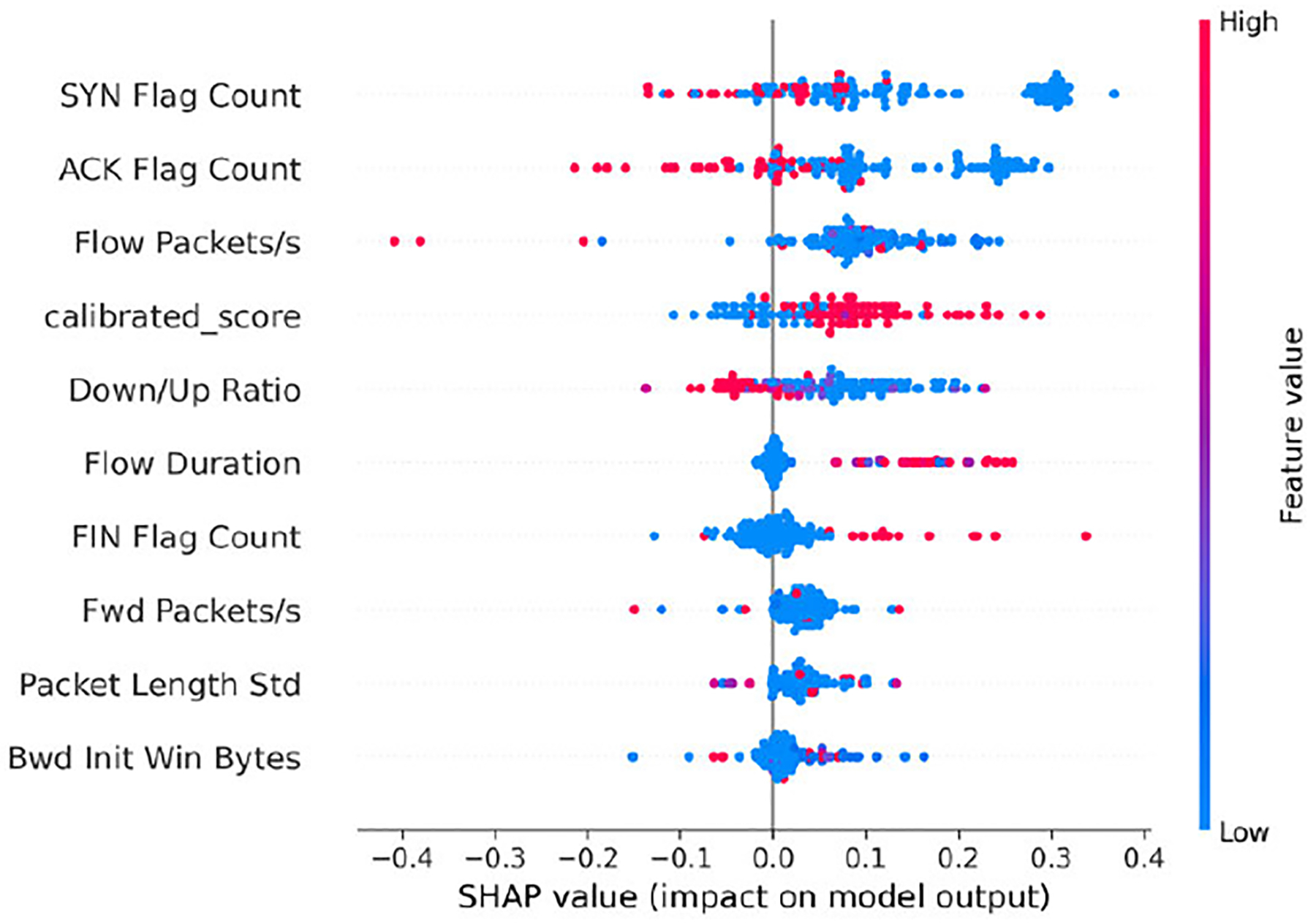
SHAP beeswarm plot for the Benign class (subset of test data).

**FIGURE 5.**
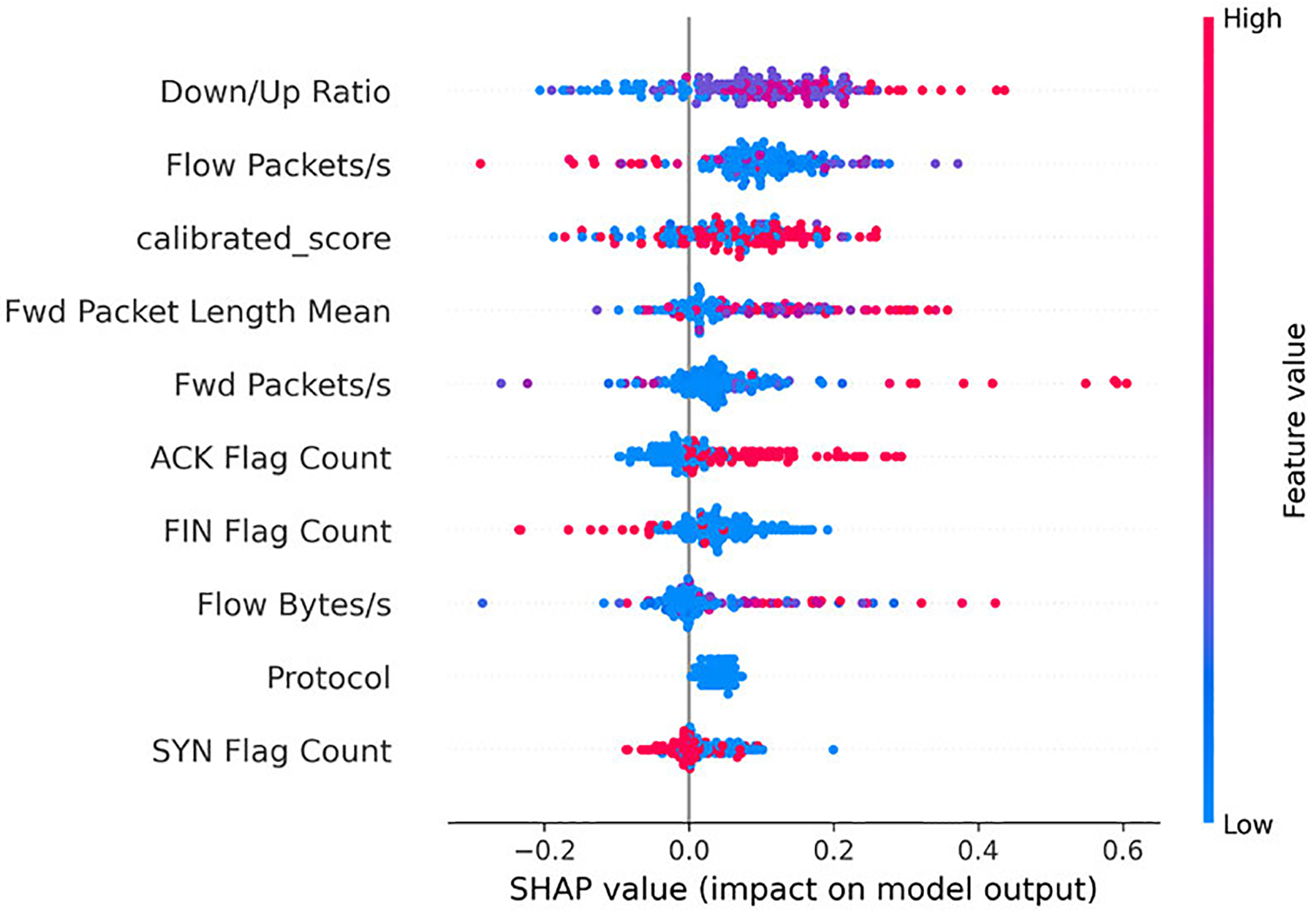
SHAP beeswarm plot for the Reconnaissance class (subset of test data).

**FIGURE 6.**
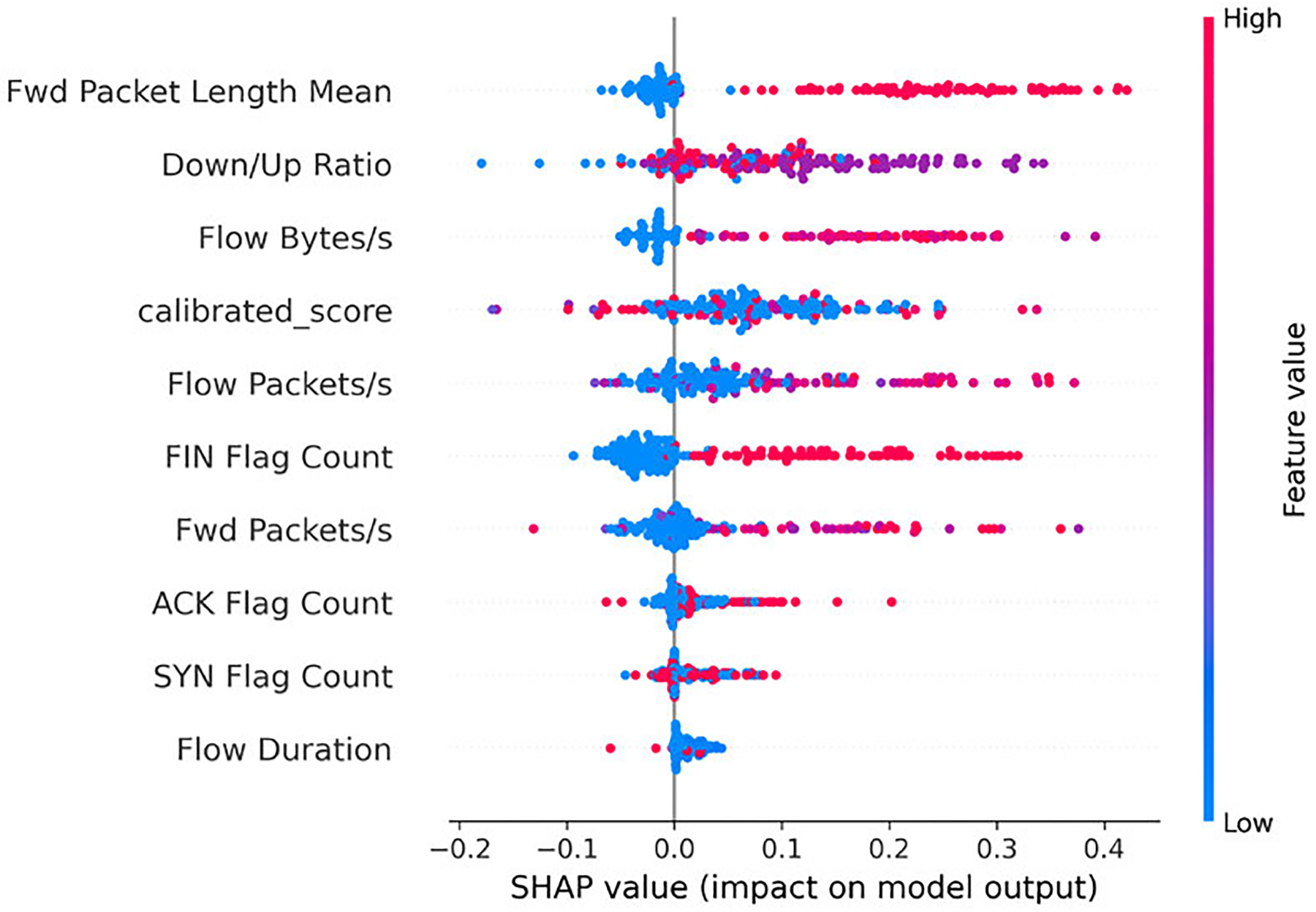
SHAP beeswarm plot for the Establish Foothold class (subset of test data).

**FIGURE 7.**
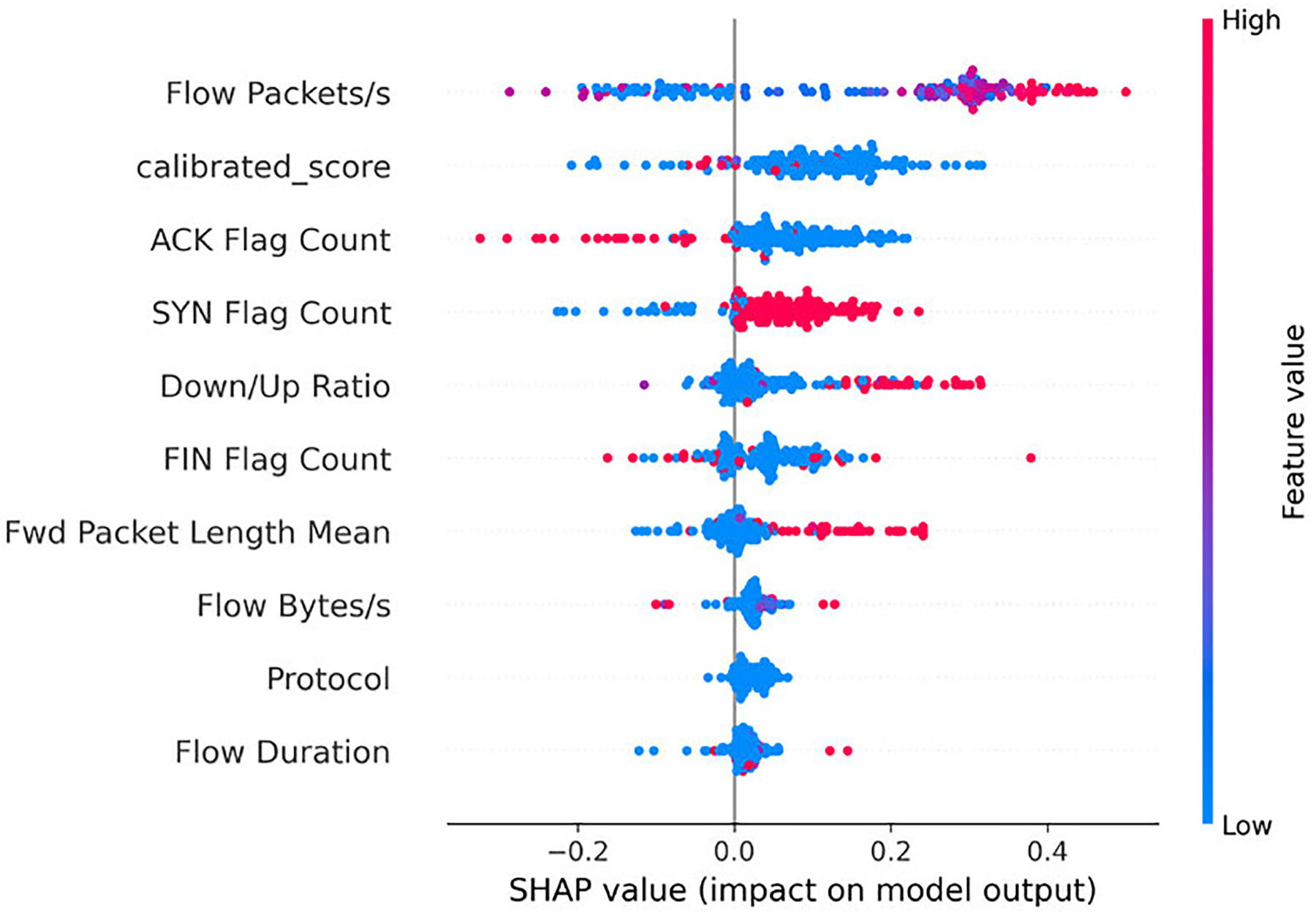
SHAP beeswarm plot for the Lateral Movement class (subset of test data).

We then examine which features most strongly characterize the stages: Benign, Reconnaissance, Establish Foothold, and Lateral Movement. Figures 4–7 present SHAP beeswarm plots, where each point corresponds to a single sample.

The horizontal axis represents the SHAP value, showing whether a feature increases or decreases the predicted probability for a given class, while the color encodes the raw feature value, highlighting how high or low feature values influence the contribution.

##### a: CLASS COMPOSITION: BENIGN

For the **Benign** stage, the traffic is characterized by short, low-rate, and lightly bidirectional exchanges. “Short” sessions are reflected by lower *Flow Duration*, while “low-rate” behavior manifests as reduced *Flow Packets/s* and *Fwd* Packets/s. The “lightly bidirectional” property indicates that although traffic exists in both directions, the downstream dominates, as captured by a low *Down/Up Ratio*. The absence of *SYN Flags* and *ACK Flags* further suggests non-TCP flows (e.g., UDP traffic), which are typically benign. These traffic patterns positively influence the prediction of the model, consistent with established Internet traffic measurements and HTTP request/response behavior [41], [42]. As illustrated in Fig. 4, the SHAP beeswarm plot confirms these patterns, showing that short durations and low packet rates are the strongest contributors to benign classification. However, it should be noted that prolonged *Flow Duration* may bias the model toward benign classification, which could represent a potential weakness when handling long probing attacks.

##### b: CLASS COMPOSITION: RECONNAISSANCE

For the *Reconnaissance* stage, traffic is characterized by low-rate, asymmetric probing behavior. ‘Low rate’ activity appears as reduced *Flow Packets/s*, since probes typically elicit only brief *ACK* replies while keeping the flow open during idle periods. The presence of an *ACK Flag* further reflects responder acknowledgment behavior. ‘Asymmetry’ is captured by a higher *Down/Up Ratio*, indicating that one direction dominates the exchange [44], [45]. In addition, the *Fwd Packet Length Mean* spans a mixture of low to high values, positioning reconnaissance traffic between benign exchanges and malicious flows bearing payloads. As shown in Fig. 5, the SHAP beeswarm plot confirms these patterns by highlighting the dominance of low packet rates and directional imbalance as key indicators of reconnaissance activity.

##### c: CLASS COMPOSITION: ESTABLISH FOOTHOLD

For the **Establish Foothold** stage, traffic is characterized by payload-bearing flows and asymmetric delivery. ‘Payload-bearing’ behavior appears as higher *Fwd Packet Length Mean* in conjunction with elevated *Flow Bytes/s*, indicating that forward segments carry substantive content beyond initial handshakes. ‘Asymmetry’ is again reflected by a higher *Down/Up Ratio*, showing that one direction dominates the exchange [47]. In addition, transmission rates tend to be elevated, as seen in increased *Flow Packets/s* and *Fwd Packets/s*, both of which frequently contribute positively to foothold classification. As illustrated in Fig. 6, the SHAP beeswarm plot highlights these patterns, confirming that payload-related characteristics and directional dominance are the most salient indicators of foothold activity.

##### d: CLASS COMPOSITION: LATERAL MOVEMENT

For the **Lateral Movement** stage, traffic is distinguished by bursty transmissions and asymmetric packet flows. ‘Bursting’ behavior is evident in higher *Flow Packets/s* and elevated *Fwd Packets/s*, indicating rapid connection attempts or short data spurts across internal hosts. ‘Asymmetry’ is reflected by a higher *Down/Up Ratio*, suggesting that one direction dominates the exchange during attacks such as credential reuse and service hopping [48], [49]. As shown in Fig. 7, the SHAP beeswarm plot highlights these patterns, confirming that bursty packet rates and directional dominance are the primary indicators of lateral movement activity.

##### e: SUMMARY OF STAGE EXPLANATIONS

Across all Stages (or Classes), SHAP analysis consistently highlights a small set of flow-level features as dominant drivers of predictions. Benign traffic is mainly characterized by short duration, low packet rates, and light bidirectional exchanges. Reconnaissance is distinguished by low-rate, asymmetric probing with intermediate packet lengths. The foothold stage is identified by payload-bearing forward flows and elevated packet rates, while lateral movement is marked by bursty transmissions and strong directional asymmetry. Taken together, these results demonstrate that SHAP not only validates the role of anomaly scores in stage-level discrimination but also provides actionable behavioral signatures that align with known APT tactics and techniques.

#### 2) LOCAL EXPLANATION

For an individual instance $x=\left(x_{1},x_{2},\ldots ,x_{p}\right)$ with p features, the model prediction can be decomposed as

(24) $$f(x)=\phi_{0}+\sum_{j=1}^{p}\phi_{j}(x),$$

where $\phi_{0}$ denotes the baseline output and $\phi_{j}(x)$ is the Shapley value assigned to feature j for instance x [5].

We illustrate local interpretability by analyzing one Benign flow and two Reconnaissance flows, showing how the global attribution patterns manifest at the instance level. Local explanations are particularly valuable when model behavior deviates from the global trends, as they highlight feature contributions that drive unexpected predictions. Such insights provide security analysts with actionable clarity, drawing attention to specific cases where the model may struggle and enabling targeted investigation of misclassifications.

##### a: BENIGN EXAMPLE

**FIGURE 8.**
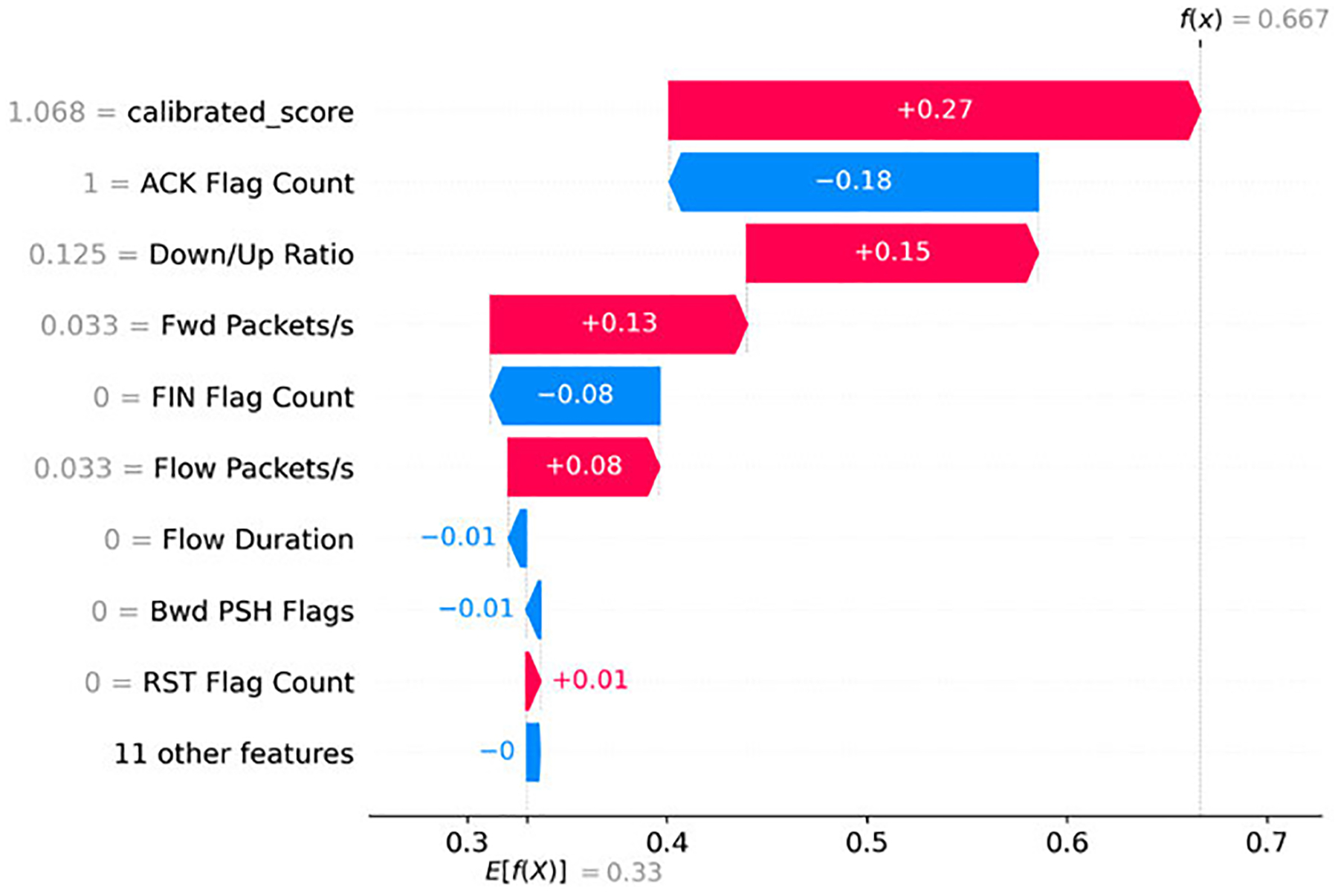
SHAP waterfall plot for a single instance in the Benign class.

Figure 8 shows a SHAP waterfall plot for our prediction model. The baseline probability is E[f(X)]=0.33, corresponding to one out of three. The final prediction is f(x)=0.667, indicating that two of the three are for the benign class.

Consistent with the global attributions observed in the Benign beeswarm plot (Fig. 4), the largest positive contributions arise from lower packet rates—*Fwd Packets/s* ≈ 0.033(+0.13) and *Flow Packets/s* ≈ 0.033(+0.08)—together with a more balanced traffic direction (*Down/Up Ratio* = 0.125, (+0.15)). These patterns are typical of brief request/response exchanges [41].

The most significant negative contributions are due to *ACK Flag Count* = 1 (−0.18) and *FIN Flag Count* = 0 (−0.08), which correspond to common TCP behavior when packets are successfully received. Despite these negative pushes, the model correctly classified the instance as Benign, primarily due to the strong positive contribution (+0.27) from the *calibrated_score*. This example underscores the central role of the calibrated anomaly score in the model’s decision-making process, aligning with our global findings.

##### b: RECONNAISSANCE EXAMPLE

**FIGURE 9.**
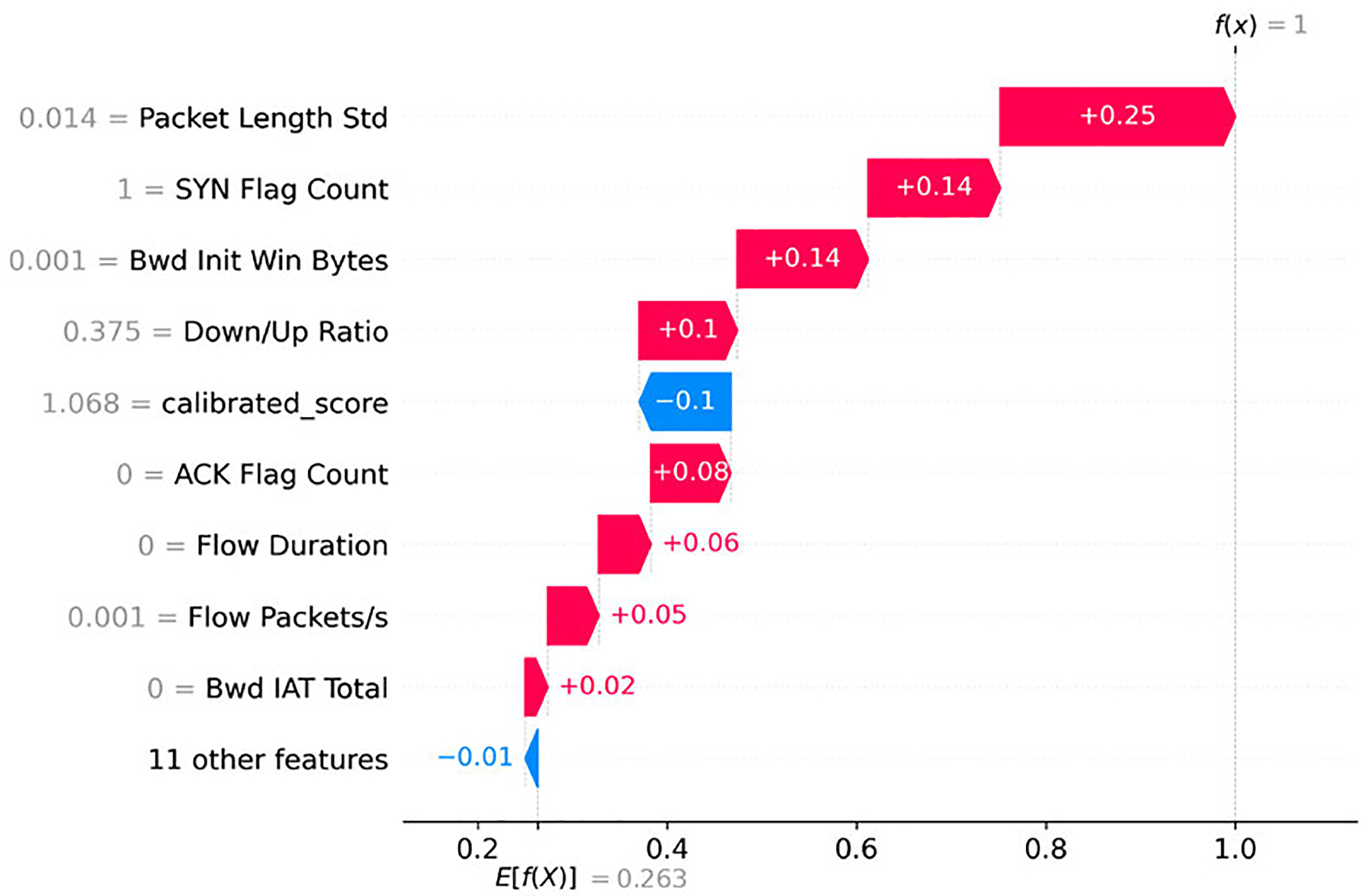
SHAP waterfall plot for an instance in the Reconnaissance class.

Figure 9 presents a SHAP waterfall plot for an instance classified as Reconnaissance. The most significant positive contributions align with typical probing behavior. A low *Packet Length Std* indicates uniform, templated packets, while near-zero *Bwd Init Win Bytes* suggests little or no advertised receive window from the responder. Short *Flow Duration* and *low Bwd Packets/s* further reveal sparse reverse traffic. Together, these features are characteristic of half-open or non-payload scans that fail to elicit full server responses. A high *Down/Up Ratio* provides additional evidence of forward-heavy traffic, a well-known indicator of probing activity [44], [46].

Interestingly, the *calibrated_score* contributes negatively in this case (−0.10), which is consistent with the global attribution pattern for Reconnaissance. This illustrates how local explanations can highlight the interaction between packet-level indicators and anomaly scores in shaping the final prediction.

##### c: INCORRECTLY CLASSIFIED EXAMPLE

**FIGURE 10.**
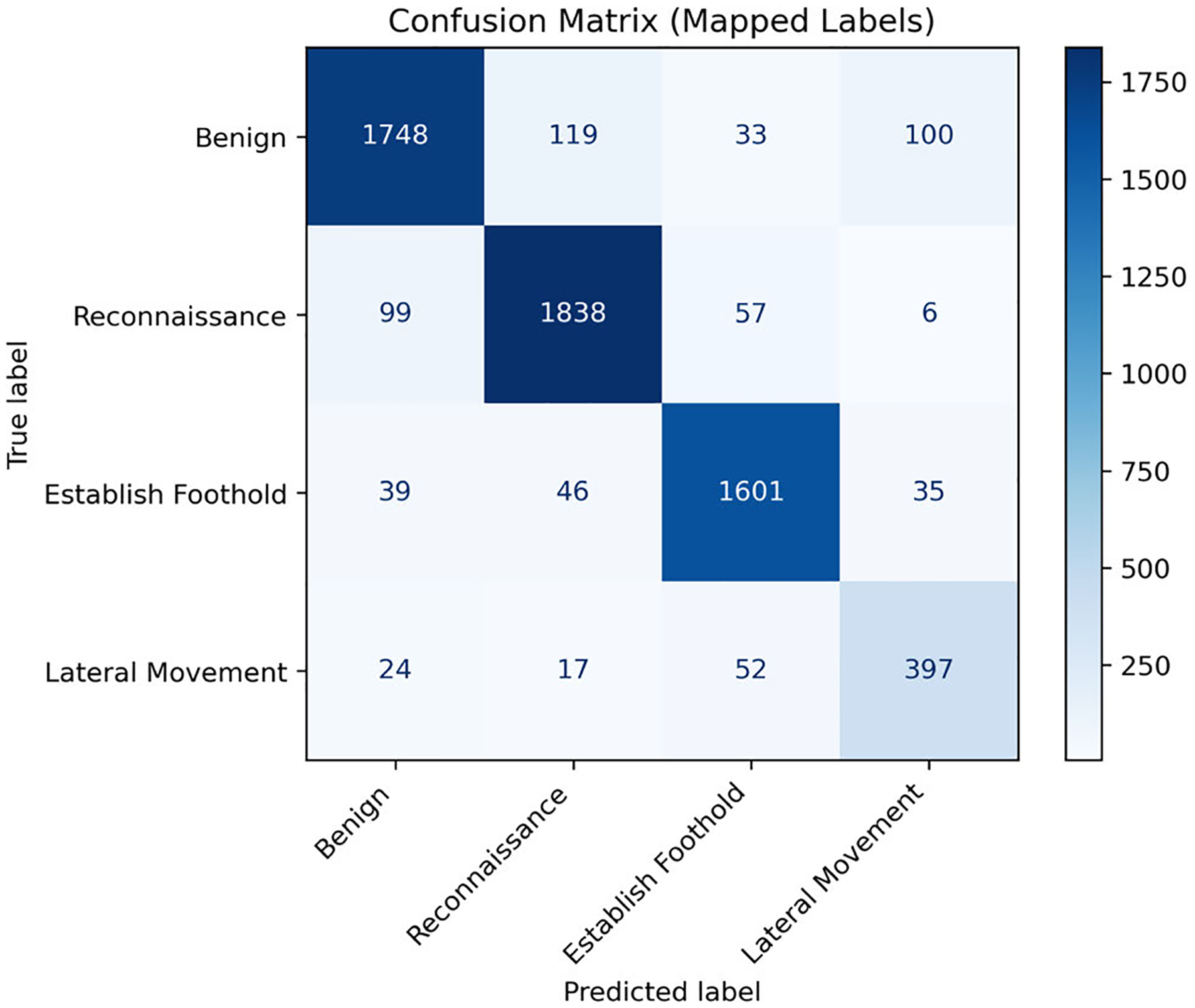
Confusion Matrix from balanced testing data.

**FIGURE 11.**
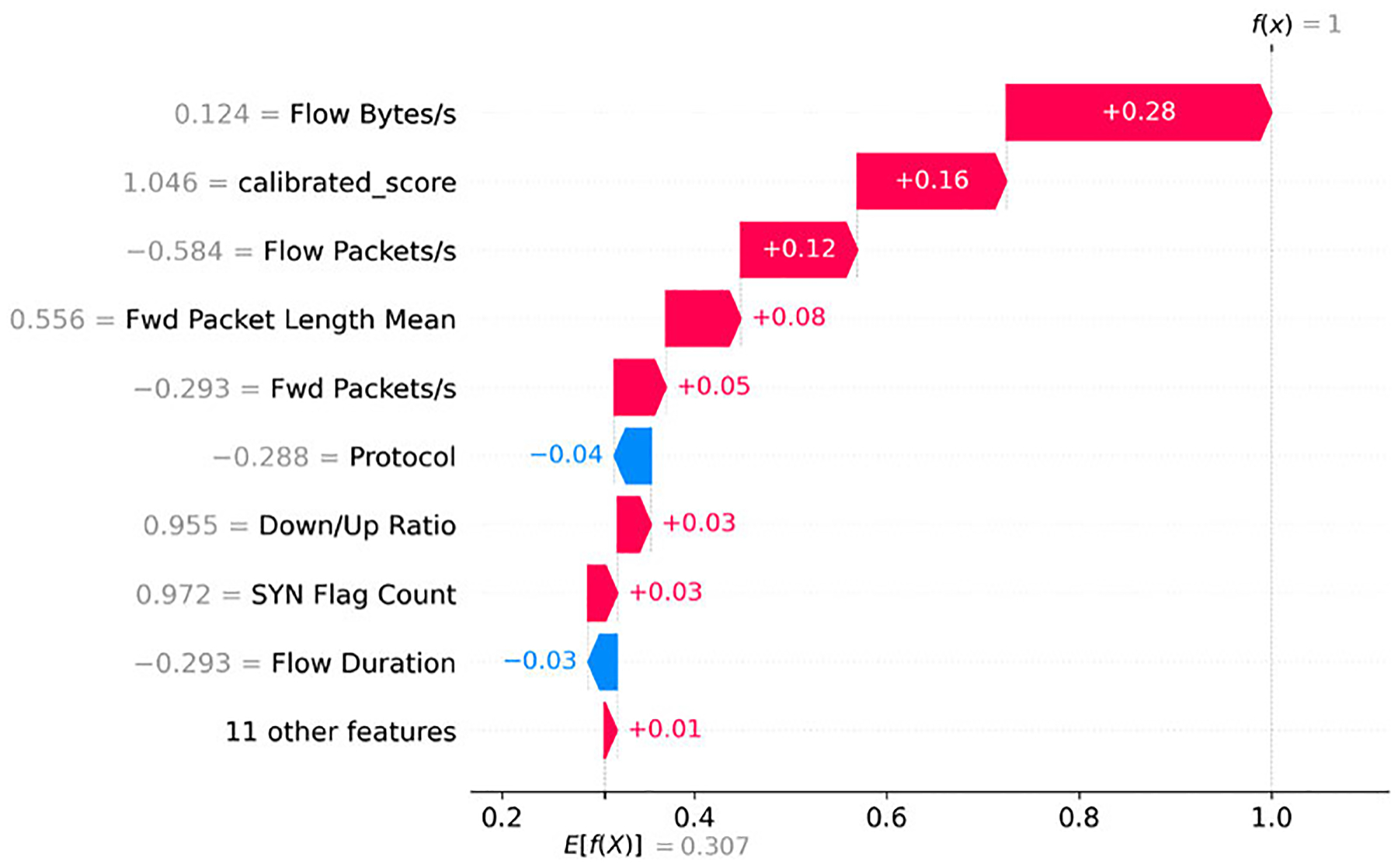
SHAP waterfall plot for a Reconnaissance instance misclassified as Benign.

Figure 11 illustrates a misclassification case where a Reconnaissance instance is incorrectly predicted as Benign. In this example, positive SHAP contributions shift the decision toward the Benign class. As also reflected in the confusion matrix (Fig. 10), Reconnaissance flows are misclassified as Benign more frequently than other attack stages. Analyzing such cases provides information on the specific weaknesses of the model, guiding improvements in APT detection strategies.

Key observations show low values of *Flow Bytes/s, Flow Packets/s*, and *Fwd Packets/s*, which are interpreted as positive indicators for Benign classification, consistent with the global pattern shown in Fig. 4. A high *calibrated_score* further reinforces this bias toward Benign. In contrast, genuine Reconnaissance traffic typically exhibits low *Flow Packets/s* and *Fwd Packets/s*, which resemble the ‘short’ and ‘low rate’ patterns characteristic of Benign flows.

Typically, higher values of *Flow Bytes/s* would contribute to a Reconnaissance prediction. However, in this misclassified case, the value is low, ultimately pushing the classification toward Benign. This suggests that Reconnaissance probes with minimal *Flow Bytes/s*, *Flow Packets/s*, and *Fwd Packets/s* are more likely to evade detection. Moreover, the elevated *calibrated_score* acts as a confounding factor, indicating that the model can be misled when Reconnaissance and Benign traffic share overlapping characteristics.

##### d: SUMMARY OF LOCAL EXPLANATIONS

Local case studies highlight how SHAP explanations provide complementary insights to global attribution patterns. For the Benign case, low packet rates and balanced traffic direction aligned with the global features of benign flows, while the calibrated anomaly score served as a decisive positive contributor. For correctly classified Reconnaissance flow, uniform packet lengths, sparse reverse traffic, and directional asymmetry reflected typical probing behavior, although the calibrated score contributed negatively in line with global tendencies. Finally, the misclassified Reconnaissance example highlighted the model’s difficulty in distinguishing low-rate probing from benign short flows, particularly when the calibrated score nudges the prediction toward the Benign class.

Together, these observations demonstrate that local explanations are crucial for comprehending both accurate and inaccurate predictions. They expose cases where feature contributions reinforce global patterns and cases where overlapping traffic characteristics lead to errors, thereby offering valuable guidance for refining the XAPT framework.

#### 3) ROLE OF THE CALIBRATED SCORE

Across all four classes, the *calibrated_score* emerges as one of the most influential features in shaping model predictions.

In the Benign class (Fig. 4), high values of *calibrated_score* are associated with positive SHAP contributions, consistently pushing predictions toward Benign. For Reconnaissance (Fig. 5), the pattern is less clearly defined. Concentrations of higher *calibrated_score* values still contribute positively, but this overlap with benign flows can mislead the model, as also reflected in the confusion matrix (Fig. 10) and illustrated in the misclassified Reconnaissance example (Fig. 11).

In contrast, the Establish Foothold class (Fig. 6) shows a more distinct trend: lower *calibrated_score* values contribute positively, reinforcing foothold predictions. For Lateral Movement (Fig. 7), this trend becomes even stronger, with clear clusters of low *calibrated_score* values producing large positive SHAP contributions. This indicates that the calibrated score captures critical metadata that informs the detection of advanced lateral movement attacks [50]. However, the confusion matrix in Fig. 10 also reveals that benign flows are sometimes misclassified as Lateral Movement. This error is likely due to the under-representation of Lateral Movement in the dataset, which biases the model toward over-attributing certain benign flows to this class.

Overall, these results highlight the pivotal role of the *calibrated_score*. By design, lower values correspond to benign traffic, whereas higher values reflect stronger anomaly signals linked to APT stages. The SHAP analyses across all four classes confirm this expected behavior, while also revealing cases where overlapping traffic patterns blur the distinction between Benign and early-stage probing. Although the *calibrated_score* occasionally introduces confusion between Benign and Reconnaissance flows, it remains the dominant driver of correct predictions across all stages. This finding validates our design choice of integrating calibrated anomaly scores into the Bayesian Network model, demonstrating that anomaly-informed features not only enhance predictive performance but also provide interpretable leverage points for security analysts.

#### 4) SUMMARY OF SHAP INSIGHTS

SHAP analyses reveal consistent global and local patterns: **Benign** flows are short, low-rate, and lightly bidirectional; **Reconnaissance** traffic shows low-rate, asymmetric probing; **Establish Foothold** is characterized by payload-bearing forward flows; and **Lateral Movement** exhibits bursty, asymmetric transmissions. Local case studies confirm these global trends while also exposing overlap regions, particularly between Benign and Reconnaissance, where the model is most uncertain.

The *calibrated_score* plays a pivotal yet nuanced role: while higher values generally align with benign classifications, lower values contribute to foothold and lateral predictions. However, overlap in low-rate probing scenarios can mislead the model. This highlights both the explanatory value and the class-specific limitations of the anomaly-informed feature, reinforcing its dual role as a strength and a challenge within the XAPT framework.

### E. SENSITIVITY ANALYSIS ON NUMBER OF PCA COMPONENTS AND CALIBRATION UNDER VARYING PCA DIMENSIONS

To evaluate the sensitivity of our model to the number of principal components ($n_{\text{components}}$), we varied the dimension of the PCA from 1 to 10 using the DAPT2020 dataset. All experiments were conducted with a fixed train/test split and consistent feature selection (top 20 ElasticNet features, excluding time-based and port features). The model used was KNN trained with calibrated scores appended.

As shown in Table 15, the results of the balanced dataset demonstrate negligible performance variation as $n_{\text{components}}$ increases. All three metrics—accuracy, macro recall, and Class 4 recall—remain unchanged, suggesting that the latent feature representations are stable and robust to PCA dimension choices.

In the unbalanced data set in Table 16, accuracy remained consistently high, but macro recall stabilized at 0.72 across all dimensions tested. Class 4 recall did not show sensitivity to the number of components and consistently reached 0.92.

These results suggest that the calibrated PCA-based score is not overly sensitive to the number of components, at least within the range tested (1)–(10). This robustness supports the generalizability of our method, indicating that moderate changes in the dimensionality of the anomaly signal do not impact downstream classifier performance.

Table 16 presents similar trends for the unbalanced dataset. Although macro recall is lower due to class imbalance, Class 4 recall remains steady at 0.92, further confirming that our calibrated anomaly score is insensitive to PCA component tuning.

To further examine the effect of calibration methods under varying PCA dimensions, we evaluated the performance of the Platt scaling method in conjunction with a KNN classifier, using the calibrated reconstruction score $h_{\text{cal}}$ as input. The number of PCA components varied from 2 to 14, while all other steps of the pipeline remained unchanged.

Table 17 summarizes accuracy, macro recall, and per-class recall across the different dimensions of the PCA. The overall accuracy peaked at 0.855 with six components and showed only modest fluctuations across the range. The macro recall ranged from 0.788 to 0.838, suggesting good stability of the classifier’s behavior with respect to the calibration input.

Of particular interest is the performance on classes 4 (lateral movement) and 6 (external control), two key stages in the APT lifecycle. Recall for class 4 remained consistently high (between 0.87 and 0.93), while class 6 showed more variation (from 0.70 to 0.77), likely due to class imbalance and the complexity of distinguishing external control behaviors. The general trend of performance indicates that the classifier is not too sensitive to the choice of PCA dimension when using calibrated scores, and the performance remains robust over a wide range.

### F. RUNTIME PERFORMANCE ANALYSIS

While the XAPT pipeline is designed for lightweight, modular deployment, its SHAP-based explainability stage can incur computational overhead. To quantify this, we conducted timing experiments comparing standard non-explainable inference with SHAP explanations under varying feature dimensions and background sizes.

Table 18 summarizes inference runtimes using top-K features selected after correlation pruning and ElasticNet selection. The non-explainable model demonstrates real-time capabilities, requiring milliseconds per sample.

Table 19 presents SHAP explanation runtimes using Kernel SHAP. As expected, the runtime grows with the background size and feature count. When K=20, runtimes increase sharply, reflecting exponential scaling behavior.

While SHAP provides high-fidelity explanations, its computational cost can be mitigated through practical engineering strategies: (1) compressing background samples via k-means or reservoir sampling; (2) reducing floating-point precision from 64-bit to 32-bit; and (3) switching to tree-based models to leverage Tree SHAP, which is more efficient than Kernel SHAP. These optimizations, coupled with cloud or GPU-based deployments, make real-time interpretability feasible in operational environments such as SIEMs or SOCs.

### G. REAL-TIME SIEM AND SOC INTEGRATION

The modular design of XAPT facilitates seamless integration into real-time security environments such as Security Information and Event Management (SIEM) systems and Security Operations Centers (SOCs). Each step in the XAPT pipeline, including PCA-based anomaly scoring, calibrated Bayesian classification, and SHAP-based explanation, can be implemented as lightweight microservices or event-driven modules within a distributed streaming architecture. For instance, incoming network events can be continuously transformed into calibrated anomaly scores and classified in near real-time, enabling SOC analysts to monitor ongoing campaigns and respond accordingly.

Integrating SHAP into SIEM and/or SOC enriches analyst decision-making by explaining why a detection was triggered, thereby enhancing trust in automated classification and streamlining triage and escalation procedures. Given SHAP’s computational cost, a practical deployment strategy is to activate explanation services on-demand via asynchronous jobs. For example, if a network event is flagged as malicious and classified as an APT stage, the associated features, SHAP background version, and model version can be logged into the SIEM. Inside the SOC, a custom action can trigger a SHAP microservice to queue explanations for those alerts, with priority scheduling ensuring that high-severity events are processed first. This strategy prevents SHAP from interfering with time-critical operations while retaining forensic value.

In large-scale environments, SHAP performance can be further improved using distributed computing, lower precision floating points, or feature summarization (e.g., via k-means background clustering). SHAP is also powerful for policy tuning. When legitimate traffic is misclassified, analysts can inspect the top SHAP contributors and revise thresholds, rules, or allowlists accordingly. Recurring feature dominance, e.g., dst_port appearing overly influential, can also guide global model calibration to reduce overfitting and feature bias.

Table 20 demonstrates how varying the number of top features (retained after ElasticNet selection) affects model performance. From here we can see models with K=10 or 15 achieve the best tradeoff between global accuracy and sensitivity to the marginalized class, *Lateral Movement*, justifying their use in production deployments where both precision and coverage matter.

## V. CONCLUSION

APTs pose serious risks due to their stealthy and persistent nature, making stage-level attribution essential for effective defense. In this work, we proposed XAPT, a framework that integrates calibrated anomaly scoring with Bayesian network classification, augmented by SHAP-based explanations for feature-level transparency. XAPT achieves accurate predictions of the APT stages while producing interpretable outputs that support the analyst’s decision-making.

Experiments on raw network traffic and meta-alert datasets show consistently high accuracy, with alerts yielding superior results over raw traffic. SHAP analysis revealed that the model relies heavily on rate- and asymmetry-based features and identified overlaps between Benign and Reconnaissance flows in low-rate scenarios that lead to misclassifications. It also underscored the pivotal yet nuanced role of the *calibrated_score*, which strengthens the stage predictions but requires careful interpretation.

Despite promising results, several limitations warrant discussion. First, the DAPT2020 dataset exhibits a substantial class imbalance, especially in later kill-chain phases, where the limited number of annotated samples constrains classifier generalization. Second, SHAP-based feature attribution introduces additional computational overhead relative to Bayesian inference alone. Although suitable for analyst triage and SOC post-processing, fully real-time deployments may require sampling, caching, or model distillation to optimize performance. Finally, our Bayesian network assumes conditional Gaussian likelihoods, which may not fully capture the distributional characteristics of flow-level and SIEM-derived features; future work may consider non-parametric or graph-structured Bayesian formulations, extend the evaluation to additional datasets with stage-level annotations, and address class imbalance, especially for Reconnaissance and Lateral Movement. Additionally, while our current implementation utilizes a trained Bayesian Network whose structure is derived via standard score-based learning, we did not explore the model’s sensitivity to alternative network structures. Comparative evaluations between fixed and learned topologies or structural priors could provide deeper insights into the reliability and generalizability of inference.

## Figures and Tables

**TABLE 1.** Examples of features from the DAPT2020 dataset.

DAPT Features	Description
*Src Port*	Source Port
*Dst Port*	Destination Port
*Protocol*	Communication Protocol
*Flow Duration*	Flow duration
*Total Fwd Packet*	Total number of packets in the forward direction
*Total Bwd Packet*	Total number of packets in the backward direction
*Total Length of Fwd Packet*	Total size of packets in forward direction
*Total Length of Bwd Packet*	Total size of packets in backward direction
*Fwd Packet Length Max*	Maximum size of packets in forward direction
*Fwd Packet Length Min*	Minimum size of packets in forward direction
*Fwd Packet Length Mean*	Average size of packets in forward direction
*Fwd Packet Length Std*	Standard deviation of packet size in forward direction
*Fwd Act Data Pkts*	Number of packets with at least 1 byte of TCP data payload in the forward direction
*Fwd Seg Size Min*	Minimum segment size observed in the forward direction
*Active Mean*	Mean time a flow was active before becoming idle
*Active Std*	Standard deviation of time a flow was active before becoming idle
*Active Max*	Maximum time a flow was active before becoming idle
*Active Min*	Minimum time a flow was active before becoming idle
*Idle Mean*	Mean time a flow was idle before becoming active
*Idle Std*	Standard deviation of time a flow was idle before becoming active
*Idle Max*	Maximum time a flow was idle before becoming active
*Idle Min*	Minimum time a flow was idle before becoming active

**TABLE 2.** Scores before and after calibration with Platt’s method for sample observations from the DAPT2020 dataset.

Index	Uncalibrated Score	Calibrated Score
0	1.859845e-06	0.990155
1	4.373830e-07	0.016706
2	1.695222e-06	0.973548
3	1.181179e-06	0.614608
4	2.678891e-07	0.005999
⋮	⋮	⋮
17334	2.475337e-07	0.005302
17335	2.545811e-06	0.999849
17336	3.609755e-07	0.010543
17337	2.425718e-07	0.005144
17338	2.815024e-07	0.006516

**TABLE 3.** Stages associated with calibrated scores for sample observations from the DAPT2020 dataset.

Index	Stage	Uncalibrated Score	Calibrated Score
0	Establish Foothold	5.358544e-07	0.212021
1	Establish Foothold	4.301933e-07	0.153976
2	Reconnaissance	5.269342e-07	0.206559
3	Establish Foothold	4.845407e-07	0.182030
4	Benign	3.582100e-07	0.122377
⋮	⋮	⋮	⋮
17334	Benign	3.560850e-07	0.121535
17335	Benign	5.875370e-07	0.245724
17336	Benign	4.308048e-07	0.154271
17337	Benign	3.562960e-07	0.121619
17338	Benign	3.577216e-07	0.122183

**TABLE 4.** Multi-class confusion matrix based on the DAPT2020 dataset (Adapted from [56]).

Actual / Predicted	Class0	Class1	Class2	Class3	Class4
**Class0**	9474	3	0	13	0
**Class1**	0	1363	12	114	0
**Class2**	0	150	1522	0	0
**Class3**	2233	346	193	363	1
**Class4**	1017	508	19	6	2

**TABLE 5.** Class-based distribution of samples and performance results based on the DAPT2020 dataset; the metrics were computed by categorizing the observations from each class (Adapted from [56]).

Metrics	0	1	2	3	4
*Total samples and ratios*	12,724 (73.38%)	2,370 (13.67%)	1,746 (10.07%)	496 (2.86%)	3 (0.02%)
*Precision (%)*	74.46	57.51	**87.17**	73.19	66.67
*Recall (DR) (%)*	**99.83**	91.54	91.03	11.58	0.13
*F1 score (%)*	85.30	70.64	**89.06**	19.99	0.26
*Accuracy (%)*	81.16	93.47	**97.84**	83.24	91.05

**TABLE 6.** Average classification results for stage prediction based on the DAPT2020 dataset (Adapted from [56]).

	Accuracy (%)	Precision (%)	Recall (%)	F1 score (%)
*Macro average*	**89.35**	71.80	58.82	53.05
*Weighted average*	84.59	**73.39**	**95.27**	**81.79**

**TABLE 7.** List of the features from the Meta-alert dataset.

Features	Data type
*Alert creation time*	Time
*Alert Impact Severity*	String
*Source Address*	IP address
*Source Port*	Integer
*Target Address*	IP address
*Target Port*	Integer

**TABLE 8.** Scores using Platt’s method with the meta-alerts dataset.

Index	Uncalibrated_score	Calibrated_score
0	0.902274	1.000000e+00
1	0.002842	4.377480e-20
2	0.949989	1.000000e+00
3	0.360608	9.798502e-01
4	0.944757	1.000000e+00
⋮	⋮	⋮
52433	0.264691	1.107923e-04
52434	0.001256	3.530984e-20
52435	0.943337	1.000000e+00
52436	0.952207	1.000000e+00
52437	0.269667	2.173649e-04

**TABLE 9.** Multi-class confusion matrix for stage prediction based on the meta-alert dataset (Adapted from [56]).

Actual / Predicted	Class 1	Class 2	Class 3	Class 4
**Class 1**	29773	0	0	0
**Class 2**	0	29608	3	2
**Class 3**	0	0	29593	0
**Class 4**	0	0	0	29486

**TABLE 10.** Class-based distribution of samples and performance results based on the meta-alerts dataset. The metrics were computed by categorizing the observations from each class (Adapted from [56]).

Metrics	Class 1	Class 2	Class 3	Class 4
*Total samples*	29,773	29,608	29,596	29,488
*and ratios*	(25.13%)	(24.99%)	(24.98%)	(24.90%)
*Precision (%)*	**100**	**100**	99.98	99.99
*Recall (DR) (%)*	**100**	99.98	**100**	**100**
*F1 score (%)*	**100**	99.99	99.99	99.99
*Accuracy (%)*	**100**	99.99	99.99	99.99

**TABLE 11.** Average classification results for stage prediction based on the meta-alerts dataset (Adapted from [56]).

	Acc. (%)	Prec. (%)	Rec. (%)	F1 (%)
**Macro avg.**	99.99	99.99	99.99	99.99

**TABLE 12.** Performance comparison with related works. Results in bold indicate the best performance in each column.

Reference	Data set	Classifier	Acc.	Prec.	Rec.	F1
[30]	CTU Botnet and *Mila data*	Random Forest	94.62	80.69	85.45	83.00
[23]	Contagio and CICIDS2017	AE + VAE-Prob	86.90	60.00	88.00	71.30
[22]	CTU-13 data	CNN-LSTM	96.50	97.00	97.00	97.00
[37]	Network Simulator 3	Belief Rule Base	91.14	87.25	93.84	89.87
[38]	Real IoT data: APT29	SMOTE-RF	81.00	96.80	83.40	89.60
**Our work**	**DAPT2020**	**Bayesian Network**	84.59	73.39	95.27	81.79
**Our work**	**Meta-alert Data**	**Bayesian Network**	**99.99**	**99.99**	**99.99**	**99.99**

**TABLE 13.** Recent 2025 APT stage-detection approaches: qualitative comparison.

Method (Year)	Stage-aware	Explainable	Data Granularity	Cross-source	Probabilistic
CONTINUUM (arXiv’25) [58]	✔	○ (GNN saliency)	Graph/host	○	○
Uncertainty-Aware Stage Clf. (arXiv’25) [59]	✔	○ (uncertainty)	Logs/events	○	✔
Explainable IDS for APT Kill Chains (arXiv’25) [60]	✔	✔ (XAI)	Flow/IDS alerts	○	○
**XAPT (this work)**	✔	✔ (SHAP)	**Flow & SIEM alerts**	✔	✔ (Bayesian)

**TABLE 14.** Feature set of the Meta-alert dataset. Due to the small number of high-level categorical attributes, this dataset is not used for SHAP explainability analysis.

Features	Data type
Alert creation time	Time
Alert Impact Severity	String
Source Address	IP address
Source Port	Integer
Target Address	IP address
Target Port	Integer
Intrusion Kill Chain (IKC) step	Integer

**TABLE 15.** Performance metrics on Balanced Dataset with Varying PCA Components.

$n_{\text{components}}$	Accuracy	Macro Recall	Class 4 Recall
1	0.88	0.88	0.92
2	0.88	0.88	0.92
4	0.88	0.88	0.92
6	0.88	0.88	0.92
8	0.88	0.88	0.92
10	0.88	0.88	0.92

**TABLE 16.** Performance metrics on Unbalanced Dataset with Varying PCA components.

$n_{\text{components}}$	Accuracy	Macro Recall	Class 4 Recall
4	0.92	0.72	0.92
6	0.92	0.72	0.92
8	0.92	0.72	0.92
10	0.92	0.72	0.92

**TABLE 17.** Platt calibration sensitivity analysis across PCA Dimensions.

PCA Components	Accuracy	Macro Recall	Class 4 Recall	Class 6 Recall
2	0.832	0.815	0.892	0.732
4	0.826	0.805	0.888	0.704
6	**0.855**	**0.838**	**0.932**	0.754
8	0.853	0.836	0.915	0.750
10	0.824	0.818	0.898	**0.778**
12	0.797	0.795	0.875	0.770
14	0.797	0.788	0.869	0.738

**TABLE 18.** Performance runtime for non-explainable inference.

Stage	Samples	Features (K)	Time/sample (ms)	Total Time (s)
Train	22,844	10	0.0019	0.043
Test	6,211	10	0.0374	0.2322
Train	22,844	15	0.0026	0.0584
Test	6,211	15	0.0328	0.2039
Train	22,844	20	0.011	0.0246
Test	6,211	20	0.0631	0.3921

**TABLE 19.** Runtime for Kernel SHAP across background sizes and feature counts.

Features (K)	Background Size (B)	Time/sample (s)
10	400	8.25
10	800	16.24
10	1600	32.31
15	400	12.57
15	800	25.10
15	1600	67.71
20	400	40.28
20	800	236.63
20	1600	570.18
20	3200	10967.05

**TABLE 20.** Accuracy metrics based on varying number of features (K) from ElasticNet (5)-Fold CV).

Number of Features (K)	Accuracy	Macro-Recall	Macro-F1	Lateral Movement Recall
5	0.8585	0.8515	0.8579	0.8184
10	0.9231	0.9071	0.9101	0.8333
15	0.9229	0.9067	0.9094	0.8322
20	0.9117	0.8792	0.8821	0.7307
35	0.9095	0.8810	0.8809	0.7506
